# Democratized Discovery of Microsclerodermin F as an Immunophilin Ligand

**DOI:** 10.3390/md23090336

**Published:** 2025-08-24

**Authors:** Manfred Auer, Malcolm D. Walkinshaw, Jacqueline Dornan, Nhan T. Pham, Xinru Xue, Miaomiao Liu, Ronald J. Quinn, Eric M. Ross, Abimael D. Rodríguez, James J. La Clair

**Affiliations:** 1 Xenobe Research Institute, P.O. Box 3052, San Diego, CA 92163-1052, USA; eross@salk.edu; 2 School of Biological Sciences, University of Edinburgh, The King’s Buildings, Edinburgh EH9 3BF, UK; 3 School of Biological Sciences, University of Edinburgh, Michael Swann Building, Max Born Crescent, Edinburgh EH9 3JR, UK; 4 Institute for Regeneration and Repair, College of Medicine and Veterinary Medicine, University of Edinburgh, 4-5 Little France Drive, Edinburgh EH16 4UU, UK; 5 Institute for Biomedicine and Glycomics, Griffith University, Brisbane, QLD 4111, Australia; 6 Molecular Sciences Research Center, University of Puerto Rico, 1390 Ponce de León Avenue, San Juan 00926, Puerto Rico

**Keywords:** democratization, natural products, immunophilin, affinity, drug discovery

## Abstract

While immunophilins are well-recognized therapeutic targets, several members of this family of peptidyl-proline isomerases (PPIases) have yet to be subjected to ligand discovery efforts. In this study, we demonstrate a cost-effective means to identify ligands to the insufficiently investigated two-domain PPIase human Cyclophilin40 (Cyp40). Central to this effort was the use of beads, wherein a confocal nanoscanning (CONA) approach was used to rapidly probe candidates. Here, we describe how one can adapt the physical nature of microsized beads as a means to strategically reduce cost and ultimately make the discovery of small molecule hit and lead compounds more accessible to everyone irrespective of financial status (democratization).

## 1. Introduction

Ever since the first carbon base metabolites were isolated, humanity has been fascinated by their ability to regulate disease with remarkable levels of precision [1,2,3]. It is no wonder that our fascination with these molecules has led to an extremely technologically sophisticated set of tools for their discovery. These approaches are profoundly expensive, leaving this facet of life only to those with extreme wealth. We challenged this trend by discovering an active natural product hit compound with democratized expenses, less than $2500 (all funds are in USD). Such challenges usually catalyzed by a prize have led to remarkable outcomes in terms of global access to computers (One Laptop per Child) [4,5], genome sequencing [6,7], as well as global challenges such as the X-prize (https://www.xprize.org). The fact that nearly all of the top 200 drugs sold have revenues above 2 billion USD (see further discussion in the Supporting Information) [8,9], has not helped motivate the discovery of cost-effective strategies.

With financial support from the National Institutes of Health, we mapped a path which engaged both frugality and simplicity. The challenge remained that the modern pipelines for discovery including data-driven compound-mapping [10,11,12] bioactivity and biosynthetic guided [13,14], and genome mining [15,16] approaches, require multiple steps of independent experimentation. With our approach, a complex myriad of chemical isolation, spectroscopic measurements, and data analyses, are merged harmoniously with detailed biological assays reporting on target identification (mode/mechanism of action, MOA) and engagement (binding constants/parameters and selectivity). In essence, all of these approaches involve three steps: identification, confirmation and validation, each of which has its own costs. We turned our focus to see if we could complete the first two steps with the goal of spending less than $2500. To our knowledge, no methods exist at this price (see further discussion in the Supporting Information). Our strategy to solving this puzzle was to allow the molecules to freely solve these tasks with little input from us; in essence to let the compounds do the work. To do this we turned to a tool that enabled us to ‘pixelate’ target protein ligation.

**Figure 1:**
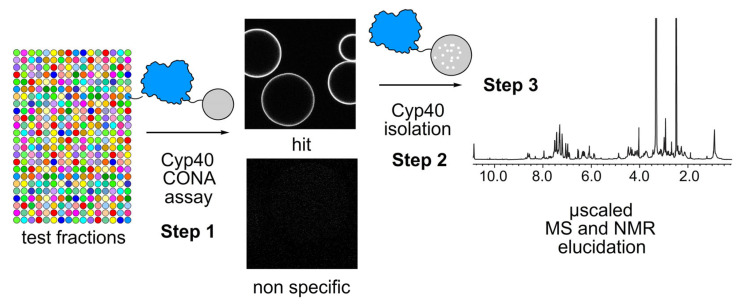
Schematic representation of the dual bead-based strategy that unites natural product identification and isolation into one experiment. (**Step 1**) CONA using solid beads was implemented to screen a panel of natural product extracts to find wells that contained fluorescent compounds that bound to Cyp40 (NatProd-CONA). Exemplary images shown after Step 1 depict fluorescence rings/halos on the outside of the bead (circles) from a hit (**top**) and non-specific binders (**bottom**). (**Step 2**) This was then followed by isolation of the binding compound using Cyp40 covalently attached to porous affinity beads. (**Step 3**) Fluorescent binders obtained from Cyp40-affinity purification are then structurally elucidated by nuclear magnetic resonance (NMR) spectroscopy and mass spectrometry (MS).

Beginning with studies on the cancer target protein survivin [17], we quickly realized the potential of a two-step two-bead-based method, shown schematically in Figure 1, to guide compound identification, however, the high level of repeats encountered required for accurate screening increased the costs above the $2500 goal. In the work leading to this publication, we show how the use of porous and solid beads offers two different yet important properties: the porous bead material was good at collecting compounds while the non-porous bead context was advantageous for structural evaluation.

We turned to an important family of therapeutic targets namely the cyclophilins [18,19]. Cyclophilins and FK506-binding proteins (FKBPs) [20,21] are members of a large class of Peptidyl-Proline Isomerases (PPIases) [22]. PPIases play a crucial role in protein folding as well as other cellular processes by catalyzing the isomerizing the *cis*- and *trans*-conformations within peptide bonds involving proline residues. This family of enzymes play a central role in modern medicine with clinical applications in immunosuppression [23], infectious disease [24,25,26], and chemotherapy [27]. Cyp40 is a unique bifunctional drug target comprised of two major domains defined by a N-terminal cyclophilin domain and a C-terminal tetratricopeptide repeat (TPR) domains. Identification of small molecules that target either domain remains an ongoing challenge for drug discovery. Although decades of effort have revealed the remarkable structural features of immunophilin enzymes [27], several members of this family, including Cyp40 [28,29], have yet to meet the critical level of therapeutic examination. Using recombinant methods, we were able to produce mg quantities of Cyp40 at $687/mg.

## 2. Results and Discussion

**Figure 2:**
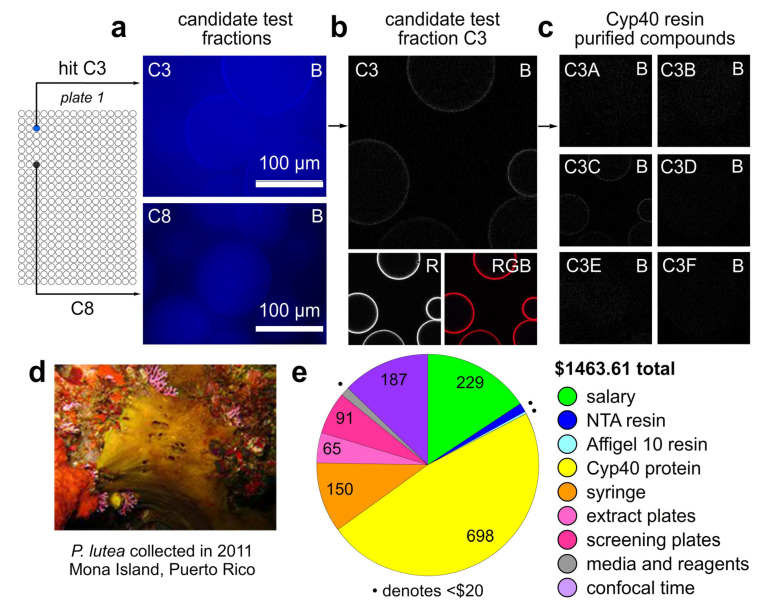
CONA analyses. (**a**) Low resolution images of a positive (C3) and exemplary negative (C8) wells taken on a conventional fluorescent microscope (Invitrogen EVOS, Carlsbad, CA, USA). As these images, clearly showed target binding but were difficult to interpret regarding affinity and binding specificity, we turned to confocal microscopy. (**b**) Complementary images collected on the positive (C3) beads using a confocal microscope (Leica SP8, Wetzlar, Germany). Binding of a fluorescent ligand was then identified by complementary fluorescent rings/halos around each bead as observed for the blue channel in well C3 (**top**). We then used Cyp40 containing <5% labelling with an orthogonal fluorescent dye, Alexa Fluor 647. Here, the overlay between the ligand and protein was readily seen in the full color image (**bottom** right) indicating that both the protein and its fluorescent ligand (hit) were present on the bead surface. This was readily distinguished from non-specific binding which appeared by fluorescence throughout the bead. (**c**) Use of CONA to track the affinity purification of the fluorescent Cyp40 binder in (**b**). Images depicting elutions (6 total) of the ligand from the beads using a gradient of water:ethanol (100:0 in A, 80:20 in B, 50:50 in C, 40:60 in D, 20:80 in E and 0:100 in F). The fluorescent ligand was observed in elution C. (**d**) Image of the sponge collected and used in this study. (**e**) Cost analysis for this study. Costs were developed using current minimum wage ($15.50/h, California 2023). Cost of materials were collected at the time of experimentation and are described in the manuscript text. Enlarged images of the beads are provided in the Supporting Figure S3.

We began our studies by applying the CONA assay to identify hit extracts and then used affinity methods to isolate Cyp40 binders from CONA active fractions (Figure 1). Leveraging prior studies [17], we turned our attention to identify fluorescent materials as they not only eased evaluation but also could be used as tool compounds for assay development. As shown in Figure 1, we began by evaluating three 384 well plates that contained fluorescent extracts (those that fluoresced under black light, excitation at 254 nm). It took 2.3 h (salary calculated by California minimum wage of $15.50/h in 2023) to complete this effort using a 100 µL Hamilton syringe (cost tabulation in Figure 2e) with a total cost of $250.45 (including the cost for the syringe). Use of this syringe was far less than a pipettor and modest washing was required to rapidly load the plate.

We then prepared HisPur Ni-NTA resin (ThermoFisher Scientific, Waltham, MA, USA) containing surface bound Cyp40. To ensure that the loading was conducted correctly and sufficient Cyp40 was on each bead, we added 0.05 equivalents of Alexa Fluor 647 NHS ester to Cyp40 (1 eq) to generate a 4.7 ± 0.2% labeled protein (Supporting Information). A total of 1.5 mL was prepared for this study. Using a gel loading tip (experimental procedures in the Supporting Information), ~1 µL of resin (see further discussion in the Supporting Information) was then loaded into each well of a glass bottom 384 well plate (142761, ThermoFisher Scientific) containing 37 µL of PBS pH 7.4 to provide a layer of HisPur Ni-NTA beads in each well. We then added 2 µL of extract to each well using a microliter syringe.

We then evaluated the assay using fluorescence imaging. As shown in Figure 2a, using fluorescence imaging on a conventional microscope (Invitrogen EVOS) we were able to suggest potential hits as illustrated by wells C3 and C8 on plate 1. Potential hit wells were further investigated by imaging on a confocal laser scanning microscope. To further the rigor of the assay, we conducted a partial labeling of Cyp40 with <5% of Alexa Fluor 647 fluorescence (bottom left, Figure 2b). This allowed us to ensure that the beads were sufficiently loaded with His_6_-Cyp40 in the red channel and then screen for hits in the outside of the Alexa Fluor 647 spectral window. Across the three plates, one hit (C3) was found in Plate 1 (Figure 2) over three repetitions of the experiment. The images shown in Figure 2b depict the hit in the blue channel (B, Figure 2b) which localized with the red fluorescence from the partially tagged His_6_-Cyp40 in the red channel (R, Figure 2b).

Using current online prices (experiment conducted on 26 October 2023) and minimum wage (2023 in California) to calculate, this process took 8.2 h to complete ($127.10) and required HisPur Ni-NTA resin ($19.15 for 1.5 mL), His_6_-Cyp40 ($346.16 for 0.5 mg), Alexa Fluor 647 NHS ester ($0.30 for 0.7 µg), Amicon UFC5003 concentrator ($8.08) and glass bottomed 384 well plates ($91.00 for 3), and PBS buffer ($1.39). A total 4 h of confocal time was used to conduct this experiment adding $124.64 at $31.16/h for a total cost of $717.81 to complete this screen. We conducted this experiment three times and the highest costs were reported in Figure 2d.

With a potential hit detected, we shifted our efforts to identify the active component in well C3. Here, we turned to the affinity of the interaction to Cyp40 as the vehicle for purification. The key to this was switching our methods from surface-active beads (beads from HisPur Ni-NTA resin used in (Figure 2a) to different bead materials for compound isolation. The use of a porous beads (Affi-Gel 10, Bio-Rad, Hercules, CA, USA) allowed us to reduce the material requirements as well as use covalently attached Cyp40 to ensure viable capture and release. We prepared 0.5 mL of Affi-Gel 10 containing 1.25 mg/mL of covalently attached Cyp40 in a 2 mL disposable column (Pierce 29920, Thermo Fisher Scientific). Fortunately, the loading of Cyp40 was very efficient in PBS pH 7.4 with over 99% loaded within 30 min at 4 °C. After capping with ethanolamine and washing three times with 5 mL of PBS pH 7.4 buffer, the beads were ready for use. A 100 µL aliquot of the extract in DMSO was diluted into PBS pH 7.4 buffer (1400 µL) and this solution was passed through the beads four times. Directly after loading, the beads were eluted with six sequential washes (1 mL) where the percentage of ethanol increased over each wash (A at 10% *v*/*v* EtOH; B at 25% *v*/*v* EtOH; C at 40% *v*/*v* EtOH; D at 55% *v*/*v* EtOH; E at 70% *v*/*v* EtOH; and F at 85% *v*/*v* EtOH). The washes were transferred to ½ dram glass vials (Glass Vials V1235CTFE, Fisher Scientific, Hampton, NH, USA) and dried by airflow. Prior to drying, fluorescence was observed in fraction C by UV illumination (254 nm), suggesting that the active component was in fraction C. We then used the same CONA system (Figure 2c) to confirm that fraction C was able to replicate the localization pattern from the initial test fraction (panel E07C, Figure 2c).

This purification step process took 4.3 h to complete (salary of $66.65) and required Affi-Gel 10 resin ($5.00 for 0.5 mL), His_6_-Cyp40 ($351.56 from $346.16 for affinity purification and $5.41 for CONA screening), a bead column ($2.21), ethanolamine ($0.04 for 100 µL), ½ dram glass vials ($2.65), ethanol ($3.92), HisPur Ni-NTA resin for screening ($0.30 for 0.02 mL), and PBS buffer ($0.70). A total 2 h of confocal time was used to conduct this experiment adding $62.32 at $31.16/h for a total cost of $495.31 to complete this purification process.

The total cost to complete the effort shown in Figure 2a,c was $1463.61, (Figure 2d), and therefore under our target of $2500. This was divisible into three tasks comprised of extract preparation ($250.45), CONA screening ($709.74) and affinity purification ($495.31) (Supplemental Table S1). A cost distribution analysis is provided in Figure 2d indicating that largest costs were protein ($692.72), salary ($229.40), confocal time ($186.96), the syringe ($150.00) used to perform the liquid transfers, and the assay and extract plates ($155.80) used for the assays. The remaining costs were spent on resins ($24.45) and supplies ($11.17).

**Figure 3:**
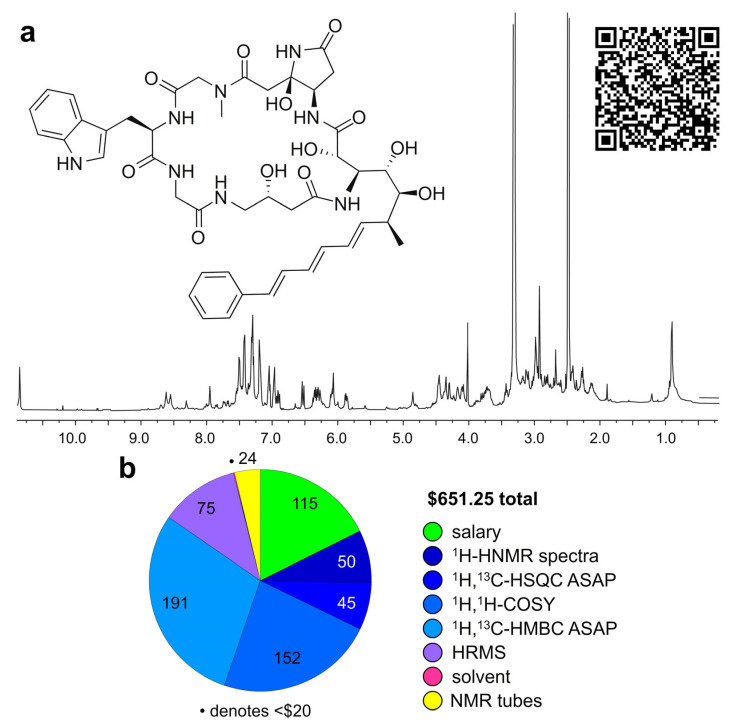
Microscale structure elucidation. (**a**) ^1^H NMR spectrum of the affinity purified C3C fraction (Figure 2c) in DMSO-*d*_6_. The material obtained from this purification was used in entirety with 15% going to HRMS analyses (Supporting Information), 5% for CONA analyses and the remaining 80% for NMR analyses. NMR analyses were collected from this sample dissolved in CD_3_OD (40 µL). (**b**) Cost required to complete the structure elucidation of the compound in C3C fraction. An extended 2D NMR data set is provided within the Supporting Information. QT code provides direct access to raw data.

**Figure 4:**
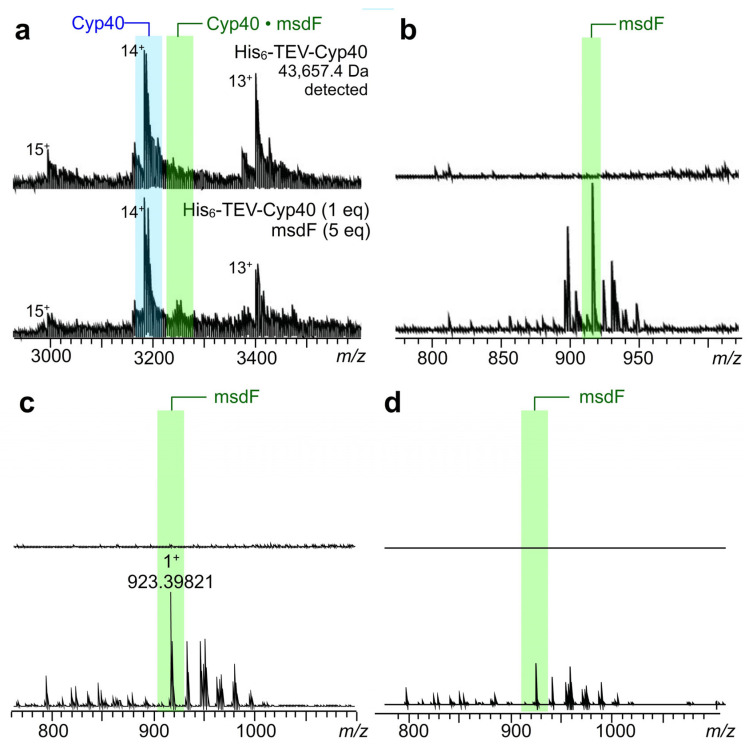
Target binding validation by Native and CIAS MS. (**a**) Native MS data collected on Cyp40 without (**top**) and with (**bottom**) microsclerodermin F (5 eq). (**b**) CIAS-MS data collected on Cyp40 without (**top**) and with (**bottom**) microsclerodermin F (5 eq). Collison induced association energy of 20 V. (**c**) CIAS-MS data collected on FKBP51 without (**top**) and with (**bottom**) 15 eq of microsclerodermin F. Collision induced association energy of 14 V. (**d**) CIAS-MS data collected on FKBP52 without (**top**) and with (**bottom**) 15 eq of microsclerodermin F. Eq denotes molar equivalents. All proteins used in this study contained a His_6_-TEV tag (see Section 4.3).

We then turned our efforts to elucidate the compound within the C3C fraction. This also had costs. The C3 fraction was obtained from a sponge (*Pachataxa lutea*, collected near Mona Island in Puerto Rico) (Figure 2d). Structure elucidation was completed by MS and NMR methods. As shown in Figure 3a, we were able to obtain sufficient purity to complete the structural elucidation process. Loading 80% of the sample into a single 40 µL solution, we were able to determine by ^1^H NMR (Figure 4a) that the affinity purified fraction contained approximately 6 µg of material (10 µg was the theoretical yield) by using ^13^C satellite analyses for standardization [30].

Our structure elucidation process began by collecting MS and HRMS analyses which returned mass *m*/*z* of 923.31 and 923.3913, respectively. Using a Google Search (Mountain View, CA, USA) to search masses *m*/*z* of 923.3910 to 923.3916 (one at a time), a link served by the University of Extremadura (Badajoz, Spain) returned a pdf file of a paper from the Faulkner laboratory describing the microsclerodermin F-I depsipeptides (923.3915 worked). Within this publication [31], the authors provided comprehensive NMR and MS data indicating that our HRMS data with a predicted formula of C_45_H_56_N_8_O_12_Na^+^ matched (delta 0.3 ppm) that reported for microsclerodermin F. At this point in the elucidation process, we already had identified a comparable peptide backbone through the use of ^1^H,^1^H-gCOSY and ^1^H,^13^C-HMBC correlations (first conducted in CD_3_OD, Supporting Information), however, access to rapid online searches considerably reduced the time to identify this material. Repeating the NMR data collection in the same solvent as the reported data (DMSO-*d*_6_) we were able to complete a detailed chemical shift and coupling constant comparison (Table S1, Figures S1 and S2, Supporting Information) and confirm that this material was microsclerodermin F.

Overall, completing the structure elucidation (Figure 3) added an additional $651.25 based on salary ($115.00 for 7.2 h) along recharge costs for instrument time at ^1^H NMR ($50.00), ^1^H,^13^C HSQC ($45.00), ^1^H,^1^H gCOSY ($150.00) and ^1^H,^13^C HMBC ($191.67), HRMS ($75.00), DMSO-*d*_6_ ($0.01) and NMR tubes ($24.38). The bulk of time spent in this study was tabulating the data (Table S1) and confirming the assignment.

At this point, we completed the process including costs for extract preparation, CONA screening, hit purification ($1463.61, Figure 2e) and structure elucidation ($651.25, Figure 3b) for a total of $2114.86. Not unexpectedly, the highest costs included instrument time, protein and salary. We believe that over time these material requirements can also be reduced. Similar cuts may also one day arise through expanding access for instrumentation for structure elucidation. Overall, we had achieved our goal of completing the first two steps (hit identification and hit confirmation) at a rate that challenges most drug discovery operations (see additional discussion in the Supporting Information). We then turned our attention to validation.

In our experience, primary screening and hit confirmation are not the most complicated facets in a compound discovery pipeline, it is rather hit validation (demonstrate target binding using a different detection technology). To address this, we turned our efforts to use methods with comparable high throughput, namely native mass spectroscopy (native MS) [32] and collision induced affinity selection mass spectrometry (CIAS-MS) [33]. Recent studies by Quinn and Liu have shown that native MS and CIAS-MS provide a facile tool for small molecule protein binding [34,35,36,37]. Using materials directly obtained from the procedure shown in Figure 3, we were able to identify binding of microsclerodermin F to Cyp40. As shown in Figure 4a, the ligated complex was readily detected in a 5:1 ratio of msdF to Cyp40. In Figure 4b, after applying collision-induced dissociation (20 V) to the formed complexes, microsclerodermin F was clearly dissociated and detected, with the major adduct being the Na^+^ ion at *m*/*z* 923, corresponding to the mass of microsclerodermin F. Similarly, the binding of msdF to FKBP51 and FKBP52 was validated by CIAS-MS (Figure 4c,d). The fact that a stronger signal was obtained for FKBP51 may suggest selectivity over FKBP52.

## 3. Conclusions

In this study, we have found that the combination of hard and porous beads with His_6_-tagged proteins can be used in a manner to discover (using HisPur Ni-NTA resin) and isolate (using Affi-Gel 10) immunophilin binding natural products. Using native mass spectroscopy for validation provides a streamlined discovery platform, one that could be subjected to cost-based democratization. Here, repurposing of tools common to other aspects of research enables one to rapidly screen for a target protein or a family of protein targets of interest. Most importantly, we find that many of the tools we use can be strategically applied to develop cost effective, democratized methods applicable to laboratories in challenged economic settings.

In Southern California, we were able to discover a fluorescent immunophilin binder for $1463.61. For an additional $651.25, we elucidated the structure of this material. By completing these tasks for $2114.86, we demonstrated a cost-effective process that might encourage scientists not only to develop data rich methods but also to remember the critical need to democratize their science. Namely, innovation is not guided by ‘data but by dollars’ and to this end many of our technological advances have shifted towards larger costs. While understood intellectually, this trend reduces the percentage of the population who have access to the growing costs with innovation. Oddly, we find that natural science unlike its artificial computational counterpart continues to drift towards costly processes.

In addition to cost, the materials and methods used in this study present minimal health risks and waste streams. The total usage for the hit discovery part of this project (Figure 2 and Figure 3) was <2 mL of beads, <10 mL of ethanol, and <200 µL of deuterated solvent. The remainder of the materials and waste from this study were aqueous media, disposable plastic plates and tubes. The use of a microliter syringe was not only a cost-effective measure but also reduced wastes. When completed, all the wastes from the screen in Figure 2 and isolation in Figure 3 were tabulated and weighed less than a cell phone (<220 g). When required, the screening plates, spin concentrators, glass vials and bead columns could be recycled. While often neglected, these variables are also an important consideration when addressing the democratization of a technology, as reduced costs also attribute to a reduction in the waste stream.

Natural Product CONA (NatProd-CONA) is a chemical and physically multiplexed, quantitative screening technique which allows a two-step identification of novel fluorescent natural products via on-bead target binding (Step 1), and identification of non-fluorescent small molecules, peptides and proteins via competitive bead-based screening (Step 2). NatProd-CONA is conducted on extracts or test fractions that have tens to hundreds of different compounds, therefore representing an element of chemical multiplexing. Physical multiplexing arises from imaging the bead samples through four (Perkin Elmer Opera Phenix) or 5-7 excitation/emission channels (Molecular Devices ImageXpress,) run in parallel on a confocal imaging plate reader. The fluorescence emission ring (halo) intensity within 5 µm of the bead’s surface represents a linear function of target binding affinity (dissociation constant, K_D_ values), of small molecular ligands at µM to nM concentrations. Therefore, NatProd-CONA a highly multiplexed screening method represents an efficient, high throughput technique for selection of the fractions/extracts for downstream isolation and structure determination by affinity-based purification, HPLC and 2D-NMR methods.

Our efforts are now focused on extending these tools towards a general screening program. We shall adapt our tools to operate without the requirement of discovering a fluorescent molecule. Prior studies by our team [17], provide strong support for this extension. We also are exploring the potential to expand to provide cost-effective strategies for hit validation (Figure 4), often the most complex aspect of the small molecule target discovery process. We realize that this study required walk-on access to State based research instrumentation, and funds were provided to complete this effort. Access to these facilities, particularly to the general public, was key to this program. We look forward to the day when we see publications that enable the discovery triad (identification, confirmation and validation) for less than $1000 USD. This should be central to scientific research, not just for natural product drug discovery.

In addition to the cost-effective aspects of this study, the discovery presented provides an important next step towards expanding access to cyclophilin inhibitors [38]. Natural products including cyclosporin (currently prescribed) [39] and sangliferins [19] that have played a key role in immunophilin chemical biological and clinical applications. Here, we identify a new cyclic peptide scaffold that can target Cyp40 and shows preliminary activity FKBP51, suggesting a new structural motif for future medicinal chemical exploration. To date, Cyp40 has not yet gained the same level of attention or validation as a drug target compared to Cyclophilin A (CypA), however, Cyp40 it is important in inflammatory, oncological and neurodegenerative diseases. Therefore, further studies are needed to clarify its potential in drug development. The discovery of ligands such as microsclerodermin F provide a critical next step to assist this. Although a subject of synthetic [40,41], chemical biological studies [42] and most recently biosynthetic gene cluster (BGC) discovery [43,44], the targets of the microsclerodermins have not yet been reported. This study provides the first evidence to their potential role as immunophilin modulators. Finally, we hope that this study inspires others to include aspects of democratization and waste reduction in their experimental design.

## 4. Materials and Methods

### 4.1. General Experimental Procedures

Deuterated NMR solvents were purchased from Cambridge Isotope Laboratories (Tewksbury, MA, USA) and were a nominal cost and not added this table. Cost of using this software was not added into the program. Procedures conducted at 4 °C were achieved by placing them in a plastic bucket containing ice.

### 4.2. Natural Product Test Fractions

Natural product test fractions screened in this program were obtained from freeze dried tissues of marine sponges, tunicates, algae and terrestrial mushrooms and plants. Extracts were prepared by soaking in EtOH or 1:1 CH_2_Cl_2_ for 12 h at 4 °C filtering and concentrating. Each extract was fractionated by loading 200 ± 50 mg of crude extract using 8 fractions A (hexanes), B (2:1 hexanes:EtOAC), C (1:1 hexanes:EtOAc), D (EtOAc), F (2:1 EtOAc:MeOH), G (1:1 EtOAc:MeOH) and H (MeOH). These extracts were dried by rotary evaporation or airflow in a rapid fashion (≤20 min) and dissolved in biological grade DMSO to provide a concentration of 15 ± 5 mg/mL. Extracts were stored at −20 °C or −80 °C until use. All samples tested were fractionated before use. Efforts are underway to develop comparable democratized methods for extract collection and fractionation.

### 4.3. Protein Production and Purification

Cyp40 (pDEST14-6H-TEV-hCyp40) was overexpressed in *E.coli* (BL21 Star DE3, Life Technologies, Thermo Fisher Scientific) and purified [45] essentially as described for CypA [46]. His_6_-TEV-FKBP51 and His_6_-TEV-FKBP52 were prepared using the same methods. In summary, proteins were purified to homogeneity by ion-metal affinity chromatography (1 mL, HiTrap IMAC FF, Cytiva, Marlborough, MA, USA) followed by gel filtration (HiLoad Superdex 200pg16/60). Fractions were pooled and concentrated using a Vivaspin 6 Centrifugal Concentrator (Sartorius, Göttingen, Germany) with a 10 kDa cut-off. This protein production was conducted at a rate of $687.40 per mg.

### 4.4. Fluorescent Protein Labeling

A stock solution of Alexa Fluor 647 NHS ester (Invitrogen) was prepared at 0.1 mg/mL in dry DMSO. An aliquot (7 µL, 0.58 nmol) of this solution was added to His_6_-Cyp40 (0.50 mg, 11.54 nmol) at 1.9 mg/mL in PBS pH 7.2 buffer at 4 °C. After incubation for 1 h at 4 °C the excess dye was removed by spin concentration using a Ultra Centrifuge Filter (UFC5003, 3 kDa MWCO, 0.5 mL sample volume) from Amicon (Miami, FL, USA) and washed 4× with an equal volume of PBS pH 7.4 (until fluorescence was not detected in the wash). Protein purity along with confirming the Alexa Fluor 647 labeling was validated by SDS PAGE gel analyses (Supporting Figure S2). The level of protein labeling was determined by using the procedure provided with the Alexa Fluor 647 labeling kit by measuring the absorbance at 280 nm (A_280_) and 650 nm (A_650_). Formulas used to calculate the degree of labeling (DOL) are provided in the Supporting Information.

### 4.5. CONA Screening

The HisPur Ni-NTA resin (ThermoFisher Scientific) was loaded with the labeled Alexa Fluor 647 tagged Cyp40 in bulk to ensure uniformity during the screening process. The entire lot prepared from Section 4.4 above was added to 1.5 mL of resin and periodically shaken (every 2–3 min to prevent settling) as it was stored at 4 °C. After 1 h, the loading process was complete as evident the loss of >95% of the fluorescence from the Alexa Fluor 647 tag (readily detected visually under a black light). Using a gel loading tip (Fisher Scientific), 1 µL of resin of HisPur Ni-NTA resin (ThermoFisher Scientific) was loaded into each well of glass bottom 384 welled plates (142761, ThermoFisher Scientific) containing 37 µL of Alexa Fluor 647 tagged Cyp40 in PBS pH 7.4 to provide a layer of HisPur Ni-NTA resin in each well. With practice the 20 µL gel loading tips can be used to load resins with a monolayer of resin at the bottom of each well. We needed to cut the tip to the right size to pull up the resin and deliver it. This required ~1 µL depending on the amount of buffer in each load. Due to cost measures, we used beads that were not sieved (use of similar sized beads through sieving eases image collection and processing) [47]. After loading each well typically contained 250 to 350 beads per well with sizes ranging 20–100 µm. We then added 2 µL of extract to each well using a microliter syringe (Hamilton 765601, Reno, NV, USA). The plates were incubated at 4°C for 30 ± 15 min. Once completed the plates were screened on a Leica SP8 with STED and Falcon at 23 °C using a 20× water immersion lens using excitation for the Alexa Fluor 647 tag with λ_ex_ at 638 nm and λ_em_ at 770 ± 5 nm to focus. The red fluorescence provided from this low-level tagging of Cyp40 provided an ideal tool to ensure homogeneous bead loading at the correct concentrations. The plate was imaged in three passes using the following wavelengths: λ_ex_ at 408 nm and λ_em_ at 450 ± 10 nm, λ_ex_ at 488 nm and λ_em_ at 590 ± 10 nm and λ_ex_ at 552 nm and λ_em_ at 660 ± 10nm. Each well was read by scanning the rows (A01–A24) by adjusting the X positioning by hand and then returning to the first well on the next row (B01). High resolution images (40×) were collected for wells with rings. A few images of the non-hit conditions were also imaged.

### 4.6. Sponge Material

The sponge *Pachataxa lutea* was collected in June 2011 during an underwater expedition near Mona Island, Puerto Rico. The sponge was frozen at −20 °C and then lyophilized. A voucher specimen (IM06-19) is stored at the Molecular Sciences Research Center, University of Puerto Rico.

### 4.7. Affinity Purification

We began by coupling the His_6_-Cyp40 on to a porous resin. Affi-Gel 10 resin (0.5 mL) from Biorad (Hercules, CA, USA) was loaded into a resin column (29920, Pierce, Thermo Fisher Scientific) and washed with cold PBS pH 7.2 buffer (3 × 0.5 mL). His_6_-Cyp40 (0.5 mg, 11.54 nmol) from a 1.9 mg/mL solution was then added to the resin and incubated at 4 °C for 30 min (set in refrigerator) with periodic shaking by hand. After this period, ethanolamine (100 µL) to cap the resin and washing (3 × 5 mL) of PBS pH 7.4 buffer, the resin was read for use. We then prepared a solution of extract (100 µL) in PBS pH 7.4 buffer (1.4 mL) and passed this solution through the resin 4 times. Directly after loading, the resin was eluted with 6 sequential washes (1 mL) with an where the percentage of ethanol increased over each wash (A at 10% *v*/*v* EtOH; B at 25% *v*/*v* EtOH; C at 40% *v*/*v* EtOH; D at 55% *v*/*v* EtOH; E at 70% *v*/*v* EtOH; and F at 85% *v*/*v* EtOH). The washes were transferred to ½ dram glass vials (Glass Vials V1235CTFE) and dried by airflow. Prior to drying, fluorescence was observed in fraction C by UV illumination (254 nm). Suggesting that the active component was in fraction C. The CONA procedure (Section 4.5 above) to confirm that fraction C was able to replicate the localization pattern from the initial test fraction (panel E07C, Figure 2c). This was conducted with 5 µL of the sample prepared herein. The solvent (aq. ethanol) was removed by airflow, and the dried fraction C was used for microscaled structure elucidation.

### 4.8. Microscaled Structure Elucidation

The material in fraction C was dissolved in CD_3_OD (50 µL, Cambridge Isotopes, Tewksbury, MA, USA). Using the 100 µL syringe, 5 µL was used to conduct the CONA assay described at the end of Section 4.7 Again, with the same loaded syringe, 40 µL of sample was loaded into a 1.7 mm NMR tube (Bruker Biospin, Billerica, MA, USA). The hole in the top of the tube was plugged with a small ball created from rolling plumbers Teflon tape (S-14666, Uline, Pleasant Prairie, WI, USA). The plugging the cap of the tube was important to ensure that the solvent did not evaporate during storage and NMR collection. The NMR tube was placed in a recycled 15 mL tube (352196, Falcon, Corning Inc., Corning, NY, USA). Recycling of these tubes is a cost-effective way to handle, process and ship NMR 1.7 mm NMR tubes. NMR data were acquired with a Bruker Avance III 600 equipped with a 1.7mm cryoprobe. Chemical shifts were referenced using the corresponding solvent signals (δ_H_ 3.31 and δ_C_ 49.0 for CD_3_OD). The NMR spectra were processed using Mestrenova (Mnova 14.3.1 Mestrelab Research, Barcelona, Spain). Costs for use of this software rated on the annual license fees were nominal and not included. The remaining contents of the vial (5 µL) was dried and used for HMRS data. To ensure that the sample was not accidentally deuterated from the CD_3_OD, we dried it down with EtOH prior (3 × 100 µL) to use.

### 4.9. Microsclerodermin F

White Powder; UV (CH_3_OH) *λ*_max_ 317 (*ε* 30,100), 332 (*ε* 23,500) nm; HR-ESI-MS *m*/*z* calcd. for C_45_H_56_N_8_O_12_ [M+Na]^+^: *m*/*z* 923.3915 (also found by Qureshi [31]), found 923.3913. A comparison of the NMR data from our sample to the literature [31] has also been conducted in the reported solvent of DMSO-*d*_6_ and is provided in Table S1. Peak shift analyses comparing published and our isolated material are provided in Figures S1 and S2. Copies of NMR spectra from this material are provided at the end of this document. Additional discussion on the assignment is provided in the Supporting Information.

### 4.10. Native MS and CIAS MS for Binding Validation

Cyp40, FKBP51 and FKBP52 were buffer-exchanged into 150 mM ammonium acetate (pH 7, 6, 6 respectively) (Sigma-Aldrich, St. Louis, MO, USA) using a Nalgene NAP-5 column (Nalgene, Rochester, NY, USA) to a final concentration of 10 µM prior to MS experiments. Before incubation with the protein, each ligand was freeze-dried and reconstituted in 1 µL of methanol. The reconstituted ligand was then mixed with 49 µL of protein solution and incubated at 4 °C for 1 h. For Cyp40, the ligand-to-protein ratio was set at 5 eq. for microsclerodermin F, while for FKBP51 and FKBP52, microsclerodermin F was used at 15 eq.

All mass spectrometry analyses were performed on a Bruker SolariX XR 12T Fourier transform ion cyclotron resonance (FT-ICR) mass spectrometer (Bruker Daltonics, Billerica, MA, USA). The electrospray ionization (ESI) source operated in direct infusion mode, employing a 500 µL Hamilton syringe with an integrated syringe pump at a flow rate of 120 µL/h. The capillary voltage was maintained at 3500 V, while the endplate offset was set to −500 V. Drying gas was supplied at 4 L/min, with a nebulizer pressure of 3 bar and a temperature of 200 °C. The source optics parameters were configured as follows: capillary exit at 200 V, deflector plate at 220 V, funnel 1 at 150 V, and skimmer 1 at 30 V. Each mass spectrum was obtained by averaging 16 transients (scans), with each transient comprising 1 million data points. Instrument operation and data acquisition were carried out using Bruker SolariX control software on a Windows platform. For native MS assays, the optical transfer frequency was set to 4 MHz, with a time-of-flight (TOF) of 1.5 ms. Mass spectra were recorded in both positive and negative ion modes, with a mass range of 100–6000 *m*/*z*. For CIAS-MS assays, the quadrupole was configured to isolate ions within an *m*/*z* range of 3000–6000. Argon served as the collision gas at a flow rate of 40%. The collision voltage was adjusted according to the experiment design. The optical transfer frequency was maintained at 4 MHz, with a TOF of 1 ms, covering a mass range of *m*/*z* 100–5000.

## Data Availability

Confocal image data is provided in raw format by email to J. J. L. (i@xenobe.org). MS and LC/MS and HPLC data is provided in raw format by email to J. J. L. (i@xenobe.org). Quick Response (QR) codes provided direct links to the raw NMR data within the NP-MRD database. All NMR data can be provided from https://np-mrd.org.

## Supplementary Materials

The following supporting information can be downloaded at: https://www.mdpi.com/article/10.3390/md23090336/s1, Figure S1: Enlarged confocal images of the resins presented in Figure 2; Figure S2: SDS PAGE gels; Figure S3: ^1^H Peak shift analysis; Figure S4: ^13^C Peak shift analysis; Figure S5: Observed ^1^H-^1^H gCOSY correlations; Table S1: Literature validation of microsclerodermin F in DMSO-d_6_.
