# Supplementary material for: Democratized Discovery of Microsclerodermin F as an Immunophilin Ligand
Source: Mar Drugs. 2025 Aug 24;23(9):336. doi: 10.3390/md23090336 (PMC12471936; doi:10.3390/md23090336)

Supporting Information

Democratized Discovery of Microsclerodermin F as an Immunophilin Ligand

Manfred Auer,[1,2,*] Malcolm D. Walkinshaw,[3] Jacqueline Dornan,[3] Nhan T. Pham,[4] Xinru Xue,[5] Miaomiao Liu,[5] Ronald J. Quinn,[5] Eric M. Ross,[1] Abimael D. Rodríguez,[6] and James J. La Clair[*,1]

[1] Dr. M. Auer, Mr. E. M. Ross, Dr. J. J. La Clair, Xenobe Research Institute, P. O. Box 3052, San Diego, CA 92163-1052, United States

[2] Dr. M. Auer, University of Edinburgh, School of Biological Sciences, The King's Buildings, Edinburgh, EH9 3BF, United Kingdom

[3] J. Dornan, Dr. M. D. Walkinshaw, University of Edinburgh, School of Biological Sciences, Michael Swann Building, Max Born Crescent, Edinburgh, EH9 3JR, United Kingdom

[4] Dr. N. T. Pham, University of Edinburgh, College of Medicine and Veterinary Medicine, Institute for Regeneration and Repair, 4-5 Little France Drive, Edinburgh, EH16 4UU, United Kingdom

[5] X. Xue, Dr. M. Liu, Dr. R. J. Quinn, Institute for Biomedicine and Glycomics, Griffith University, Brisbane, QLD 4111, Australia

[6] A. D. Rodríguez, Molecular Sciences Research Center, University of Puerto Rico, 1390 Ponce de León Avenue, San Juan, 00926, Puerto Rico

Correspondence should be directed to i@xenobe.org or manfred.auer@ed.ac.uk

Acknowledgments. This program was supported in part by funding from NIH Grant 1R21NS128597 awarded to J.J.L. and M.A. M.A. also acknowledges financial support from Medical Research Council (MRC-J54359) Strategic Grant. N.T.P. acknowledges financial support by the Dementia Research Institute (UK DRI-LA2023/2) through UK DRI Ltd., principally funded by the UK Medical Research Council.

Contents: **Page**

Additional Discussion on Cost Analyses.	S3
Formulae used for protein concentration calculations	S3
HRMS spectrum from microsclerodermin F	S4
**Figure S1**. Enlarged images of the resins depicted in Fig. 2	S5
**Figure S2.** SDS PAGE gels depicting purity and confirming Alexa 647 labeling	S6
**Figure S3.** ^{1}H Peak shift analysis	S7
**Figure S4.** ^{13}C Peak shift analysis	S8
**Figure S6.** ^{1}H,^{1}H gCOSY analysis	S9
**Table S1.** Literature validation of microsclerodermin F in DMSO-d_6.	S10-S11
^{1}H-NMR spectrum of microsclerodermin F in CD_3OD	S12
^{1}H, ^{1}H-COSY spectrum of microsclerodermin F in CD_3OD	S13
^{1}H, ^{1}H-TOCSY spectrum of microsclerodermin F in CD_3OD	S14
^{1}H, ^{13}C-HSQC spectrum of microsclerodermin F in CD_3OD	S15
^{1}H, ^{13}C-HSQC ASAP spectrum of microsclerodermin F in CD_3OD	S16
^{1}H, ^{13}C-HMBC ASAP spectrum of microsclerodermin F in CD_3OD	S17
^{1}H-NMR spectrum of microsclerodermin F in DMSO-d_6	S18
^{1}H, ^{1}H-COSY spectrum of microsclerodermin F in DMSO-d_6	S19
^{1}H, ^{13}C-HSQC ASAP spectrum of microsclerodermin F in DMSO-d_6	S20
^{1}H, ^{13}C-HMBC ASAP spectrum of microsclerodermin F in DMSO-d_6	S21

QR Codes provided on the spectral pages provided direct links to the raw data.

Additional Discussion on Cost Analyses. The authors are well aware that the novel natural product hit is only the starting point for a drug discovery process which could cost up to 2 billion USD for a drug to reach the market. However, the key point of the submitted manuscript is to offer a process which allows most laboratories, including small academic laboratories and startup ventures worldwide to be included in the early drug discovery process by providing novel chemical entities which can be offered to and taken up by the professional and experienced drug discovery teams. A recently published report [https://ftloscience.com/process-costs-drug-development/] provides an excellent tabulation of the typical costs to lead compound declaration of 353 million USD in 2022. It is fair to say that a minimum of 5-10% might be for hit selection and confirmation which would put costs for programs that get to the state described in this manuscript at 18-35 million USD. For many reasons, it is difficult to get exact numbers for a natural product drug discovery effort in industry as these costs are typically not reported on a per-project basis corporately. That noted, we do believe the methods in this program offer an expanded access for the scientific community particularly due to the fact that all materials described are commonplace and available commercially.

Formulas used to calculate the degree of labeling (DOL). The protein concentration was determined using the following formula:

$$\text{Protein concentration (mg/mL)} = \frac{[A_{280} - 0.03(A_{650})]}{A_{280} \text{ at 1 mg/mL}}$$

$$\text{Protein concentration (M)} = \frac{\text{Protein concentration (mg/mL)}}{\text{Protein molecular weight (Da)}}$$

$$\text{Degree of labeling (DOL)} = \frac{\text{A650 concentration (mg/mL)}}{239{,}000 \times \text{Protein concentration (M)}}$$

Where 239,000 $cm^{-1}M^{-1}$ is the approximate molar extinction coefficient of the Alexa Fluor 647 dye.

Using this method, we obtained DOL values of 4.7 ± 0.2% over the three repeats for the gels shown in Supporting Fig. 2.

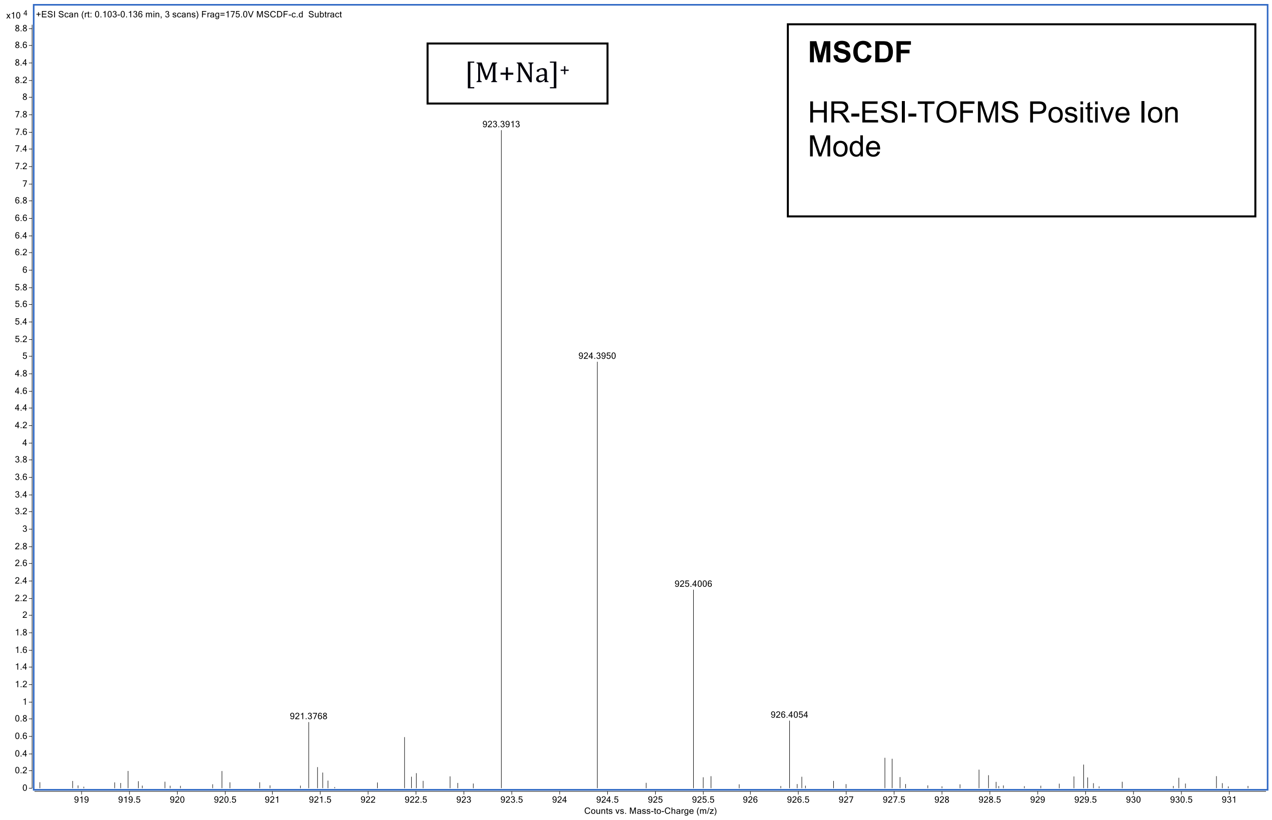

Sample MSCDF

Mass Measured	Theo. Mass	Delta (ppm)	Composition
923.3913	923.3910	0.3	$[C_{45}H_{56}N_8O_{12}Na]^+$

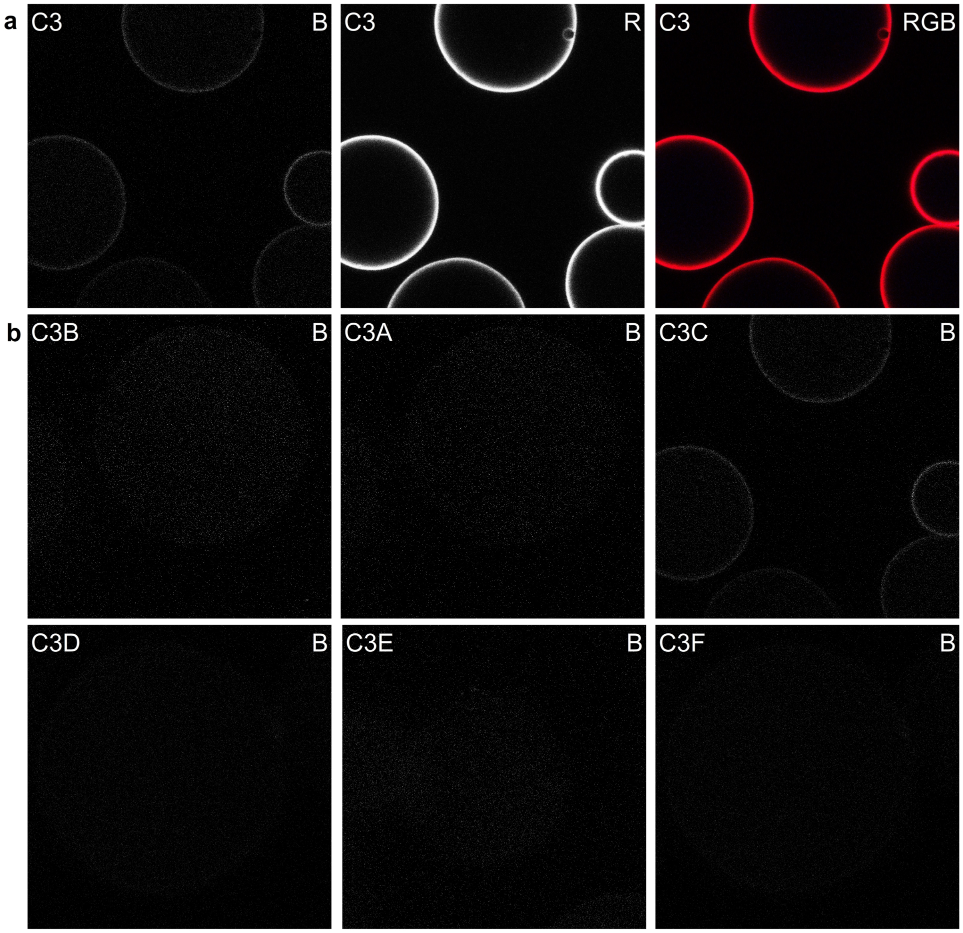

Figure S1. Enlarged confocal images of the resins presented in Fig 2 as given by **a)** the screening images in Fig. 2b and **b)** the isolation images in Fig. 2c.

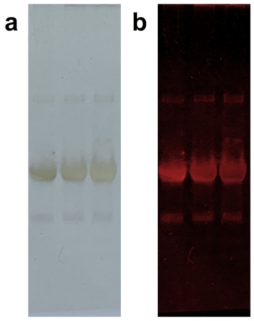

Figure S2. SDS PAGE gels depicting **a)** the purity of the Cyp40 used for these studies and **b)** confirmation of the Alexa Fluor 647 labeling. Three repetitions were conducted as shown by lanes L1 – L3. The gel was imaged in b) on a Typhoon scanner and then silver stained in a).

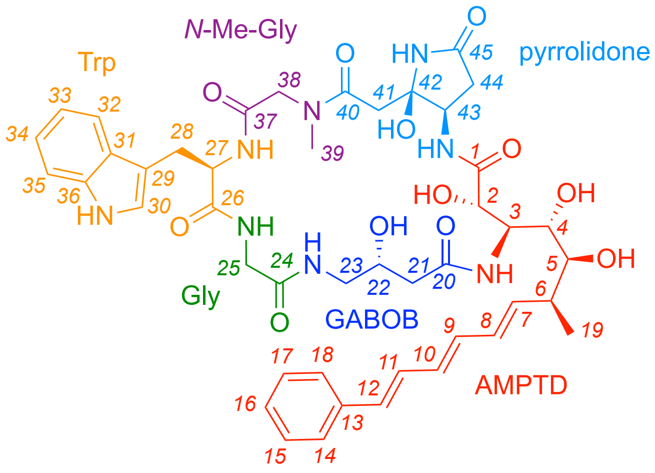

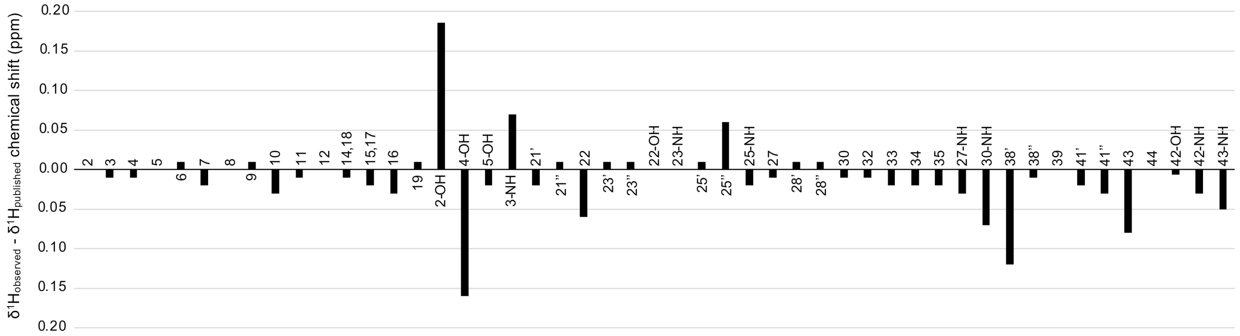

Figure S3. ^{1}H Peak shift analysis. Plot of chemical shift differences $\delta^{1}H_{observed}$ - $\delta^{1}H_{published}$ for spectral data from our sample versus that reported by Quadri.[8] Both spectra were collected in DMSO-d_6. All chemical shift deviations were within 0.2 ppm with the majority of non-exchangeable protons within δ of 0.05 ppm. Three exceptions were observed at the protons on C22 (3.79 ppm reported and 3.73 ppm observed), C38' (3.85 ppm reported and 3.83 ppm observed) and 43 (4.48 ppm reported and 3.40 ppm observed). Peaks for protons at C22 and C38' overlapped complicating their precise chemical shift assignments. The peak C43 also overlapped with the OH proton on C4 again preventing its precise chemical shift assignment. We have included spectra of our samples run in CD_3OD which was able to resolve the C22 and C38' overlap, however, had further complications due to overlap of C38 with C43 and C2. In addition, we found that the NMR spectrum of this material was very sensitive to the levels of water within the sample suggesting hydration or conformational changes on water binding.

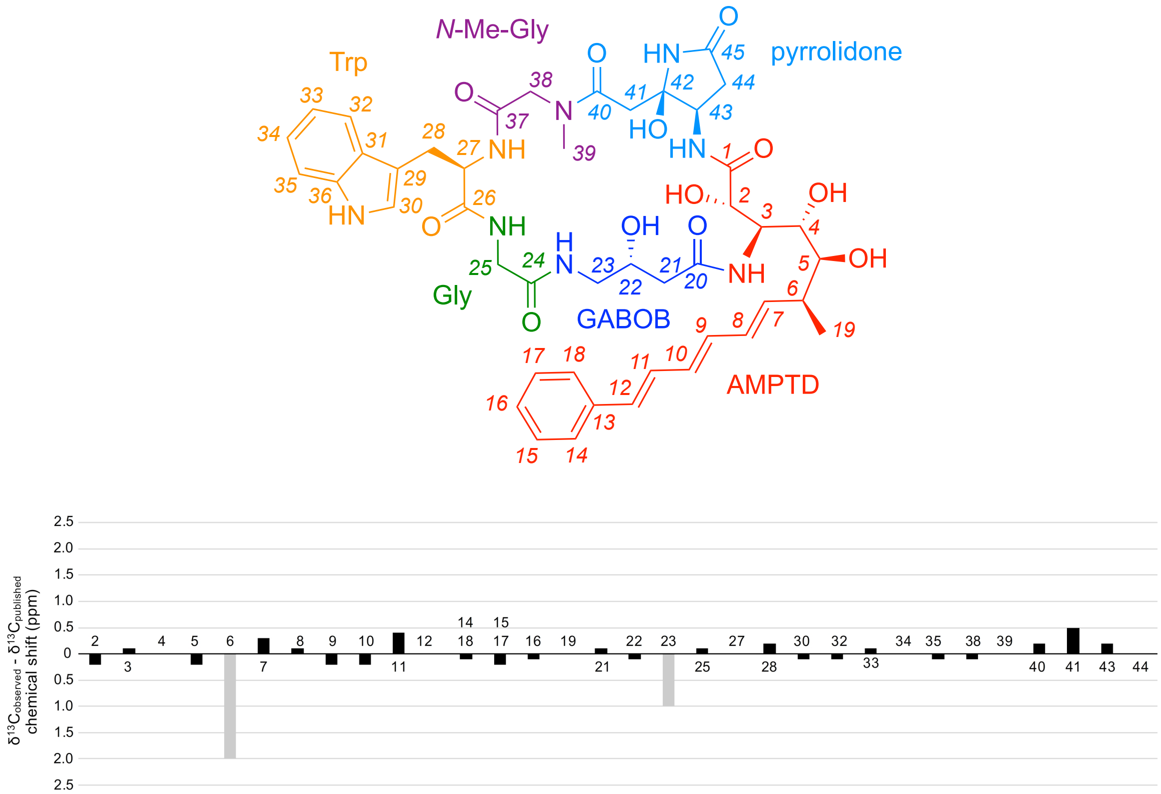

Figure S4. ^{13}C Peak shift analysis. Plot of chemical shift differences $\delta^{13}C_{publised}$ - $\delta^{13}C_{observed}$ for spectral data from our sample versus that reported by Quadri.[8] Both spectra were collected in DMSO-d_6. The majority of peaks were less than 0.5 ppm from the reported spectral data. Two exceptions existed at C6 and C23. The assignment of C6 (40.8 ppm reported and 38.8 ppm assigned) and C23' (45.0 ppm reported and 44.0, observed) were complicated due to the fact that they were underneath water peak in DMSO-d_6 complicated the precise assignment. The error observed for these two carbons should not be considered as significant and hence the bars as shaded in grey. We have included spectra of our samples run in CD_3OD to provide further support for these assignments.

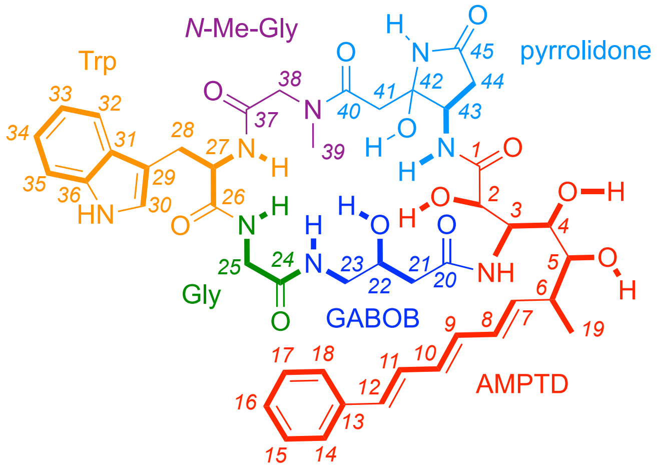

Figure S5. Observed ^{1}H-^{1}H gCOSY correlations. Key correlations are shown with bolded lines. Vicinal correlations are not shown but provided in Table S1. Correlations matched that anticipated for each amino acid residue.

Table S1. Literature validation of microsclerodermin F in DMSO-d6.

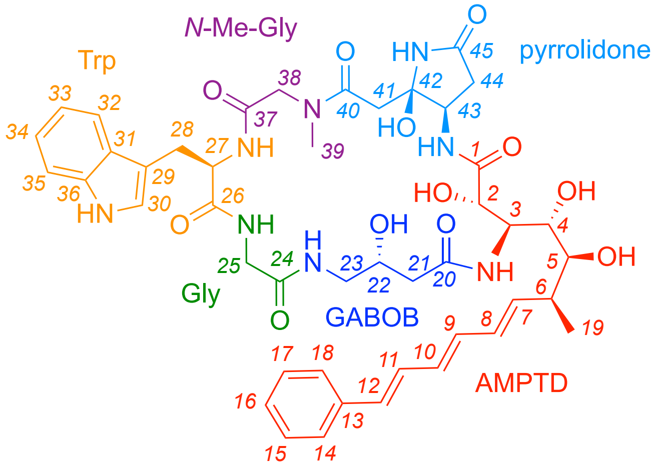

		published		observed		
amino acid	#	δH, mult (*J* in Hz)	δC, Type	δH, mult (*J* in Hz)	δC, Type[1]	^{1}H,^{1}H -gCOSY
AMPTD	1	–	172.7, C	–	NA[5]	–
AMPTD	2	4.39, d (6.0)	69.5, CH	4.39, d (5.7)	69.3, CH	2-OH
AMPTD	3	4.12, m	53.3, CH	4.11, m	53.4. CH	4w,3-NH
AMPTD	4	3.47, dd (9.5, 9,5)	68.3, CH	3.46, m	68.3, CH	3w,4-OH,
AMPTD	5	3.19, m	72.9, CH	3.19, m	72.7, CH	5-OH,6
AMPTD	6	2.44, m	40.8, CH	2.45, m	38.8, CH[7]	5,19
AMPTD	7	5.92, d (7.5)	141.0, CH	5.90, d (7.4)	141.3, CH	8
AMPTD	8	6.12, dd (7.5, 7.5)	129.0, CH	6.12, m	129.1, CH	7,9
AMPTD	9	6.37, m	134.6, CH	6.38, dd (15.0, 10.2)	134.4, CH	8,10
AMPTD	10	6.34, m	130.6, CH	6.31, m	130.4, CH	9,11
AMPTD	11	6.94, dd (15.5, 10.5)	129.6, CH	6.93, dd (15.5, 10.4)	130.0, CH	10,12
AMPTD	12	6.55, d (15.5)	131.0, CH	6.55, d (15.5)	131.0, CH	11
AMPTD	13	–	137.3, C	–	NA[5]	–
AMPTD	14,18	7.45, m	126.1, CH	7.44, d (7.4)	126.0, CH	15,16[2],17
AMPTD	15,17	7.34, m	128.8, CH	7.32, m	128.6, CH	14,16,18
AMPTD	16	7.24, m	127.3, CH	7.21, d (7.0)	127.2, CH	14[1],15,17,18[2]
AMPTD	19	0.92, d (6.5)	16.4, CH_3	0.93, d (6.9)	16.4, CH_3	6
AMPTD	2-OH	6.10, m	–	6.28, m[6]	–	2
AMPTD	4-OH	4.64, d (8.5)	–	4.48, m	–	4
AMPTD	5-OH	4.34, d (4.5)	–	4.32, d (5.1)[6]	–	5
AMPTD	3-NH	7.45, d (8.5)	–	7.52, d (7.4)	–	3
GABOB	20	–	172.9, C	–	NA[5]	–
GABOB	21’	2.18, m	41.0, CH_2	2.16, m	41.1, CH_2	21”, 22[2]
GABOB	21”	2.44, m		2.45, m		21’
GABOB	22	3.79, d (7.0)	67.2, CH	3.73, m	67.1, CH	21’[2]
GABOB	23’	2.67, m	45.0, CH	2.68, m	44.0, CH[7]	
GABOB	23”	3.44, m		3.45, m		23-NH
GABOB	22-OH	4.88, d (4.5)	–	4.88, d (5.1)	–	22

GABOB	23-NH	7.53, d (8.0)	–	7.53 d (8.0)	–	23'',25'w
Gly	24	–	169.1, C	–	NA[5]	–
Gly	25'	3.38, m	42.6, CH_2	3.39, m	42.7, CH_2	25'',25-NH
Gly	25''	3.76, m		3.82, d (16.6)		23'w,25', 25-NH
Gly	25-NH	8.59, t (6.0)		8.57, t (6.0)	–	25',25''
Trp	26	–	172.2, C	–	NA[5]	–
Trp	27	4.21, m	55.5, CH	4.20, s	55.5, CH	27-NH,28',28''
Trp	28'	2.99, m	26.2, CH_2	3.00, m	26.4, CH_2	27
Trp	28''	3.12, m		3.13, m		27
Trp	29	–	109.6	–	NA[5]	–
Trp	30	7.22, m	123.9, CH	7.21, m	123.8, CH	35w
Trp	31	–	127.2	–	NA[5]	–
Trp	32	7.53, m	118.3, CH	7.52, m	118.2, CH	33,34w,35w
Trp	33	6.99, dd (7.5, 7,5)	118.4, CH	6.97, t (7.4)	118.5, CH	32,34,35w
Trp	34	7.08, dd (7.5, 7.5)	121.0, CH	7.06, t (7.6)	121.0, CH	32w,33,35
Trp	35	7.34, m	111.5, CH	7.32, m	111.4, CH	30w
Trp	36	–	136.2, CH	–	NA[5]	–
Trp	27-NH	8.67, d (4.0)	–	8.64, d (3.8)	–	27
Trp	30-NH	10.93, s	–	10.86, s	–	
N-Me-Gly	37	–	170.3, C	–	NA[5]	–
N-Me-Gly	38'	3.85, m	49.7, CH_2	3.73, m	49.6, CH_2	38''
N-Me-Gly	38''	4.13, m		4.12, m		38'
N-Me-Gly	39	2.94, s	36.4, CH_3	2.94, s	36.4, CH_3	
Pyr[3]	40	–	170.3	–	170.5, C	–
Pyr	41'	2.72, d (17.0)	38.7, CH_2	2.70, m	39.2, CH_2	41''
Pyr	41''	2.86, d (17.0)		2.83, m		41'
Pyr	42	–	85.7, C	–	NA[5]	–
Pyr	43	4.48, dd, (9.0, 18.0)	50.6, CH	4.40, m	50.8, CH	43-NH, 44
Pyr	44	2.29, m	35.1, CH_2	2.29, m	35.1, CH_2	
Pyr	45	–	173.4	–	NA[5]	–
Pyr	42-OH	5.89, s	–	5.88, m[4]	–	
Pyr	42-NH	8.00, s	–	7.97, s	–	
Pyr	43-NH	7.57, d (8.5)	–	7.52, d, (7.0)	–	43

[1] ^{13}C NMR data was tabulated from ^{1}H,^{13}C-HSQC. We did not have sufficient sample size to obtain ^{13}C data from direct detection.

[2] w denotes a weak cross peak

[3] Pyr denotes pyrrolidone

[4] Two singlet peaks observed. This could reflect ring closed tautomers.

[5] Obtained from ^{1}H,^{13}C-HMBC data. NA denotes we could not detect this peak in the HMBC spectrum.

[6] The original publication assigned C2-OH at 4.34 ppm as C5-OH at 6.10 ppm. However, gCOSY data shows that C2 couples to C2-OH at 6.28 ppm as C5 couples to C5-OH at 4.32 ppm.

[7] Assignment of the carbon for C6 and C23 was difficult to assign due to the presence of multiple peaks and solvent at that position.

^{1}H-NMR spectrum of microsclerodermin F in CD_3OD

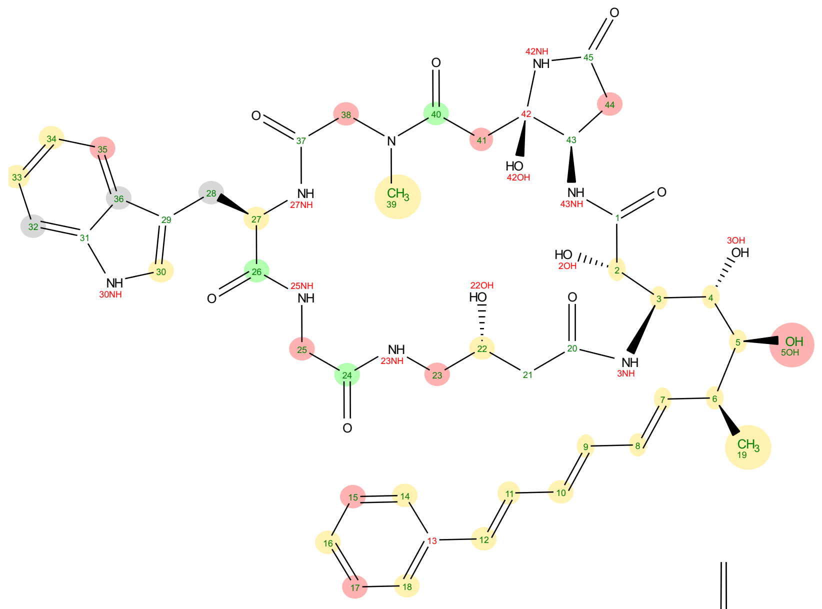

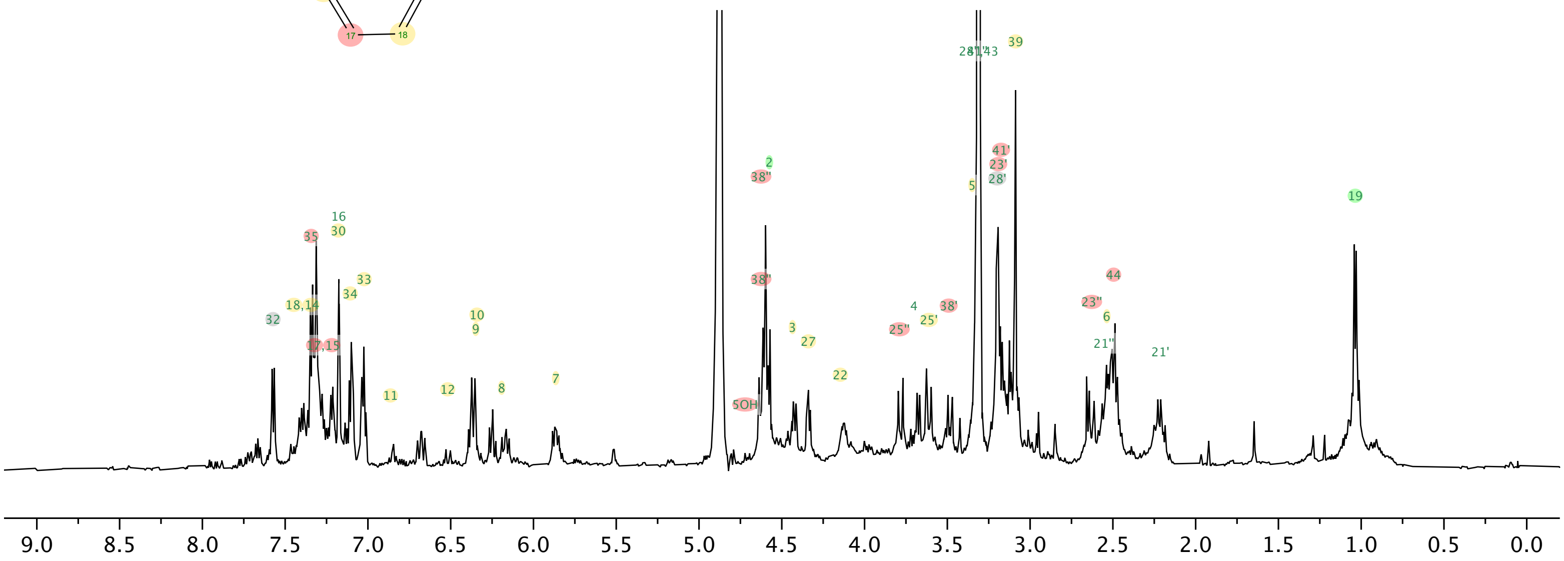

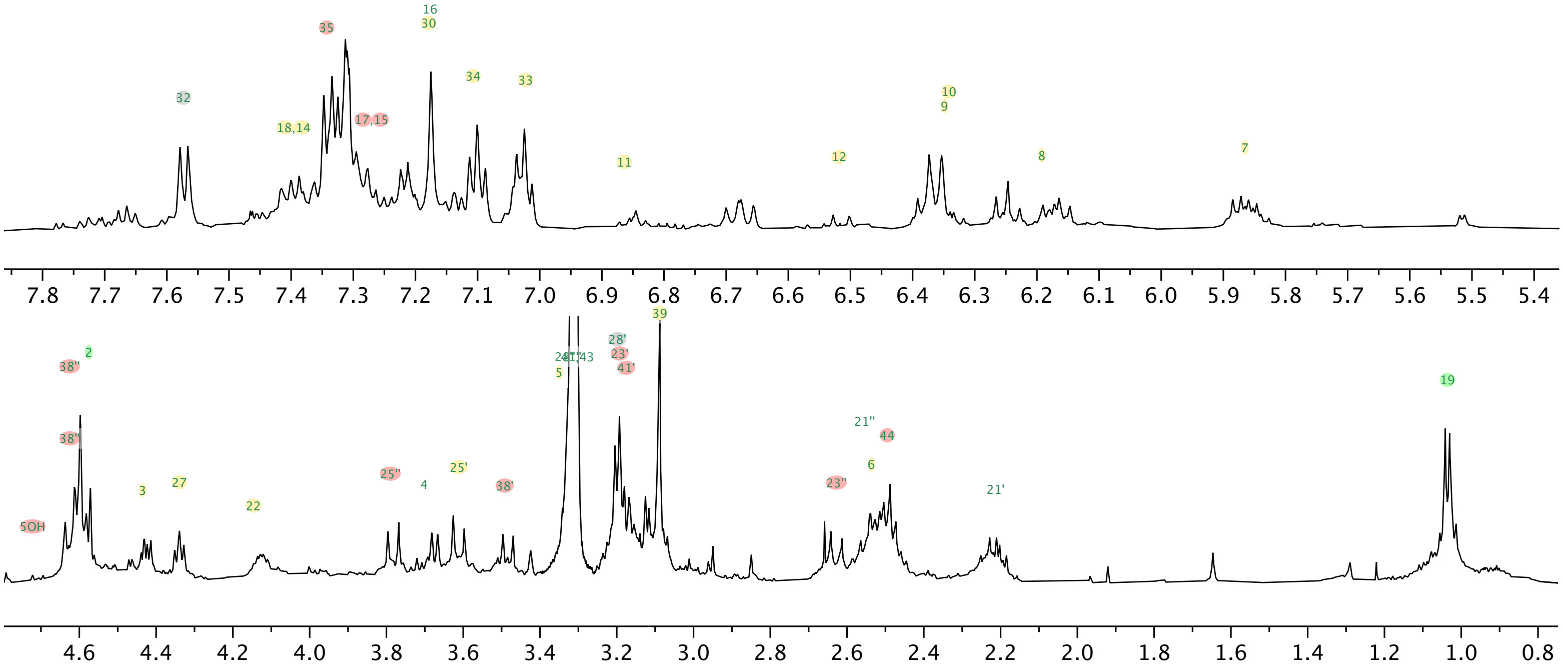

$^1H,^1H$-COSY spectrum of microsclerodermin F in CD_3OD

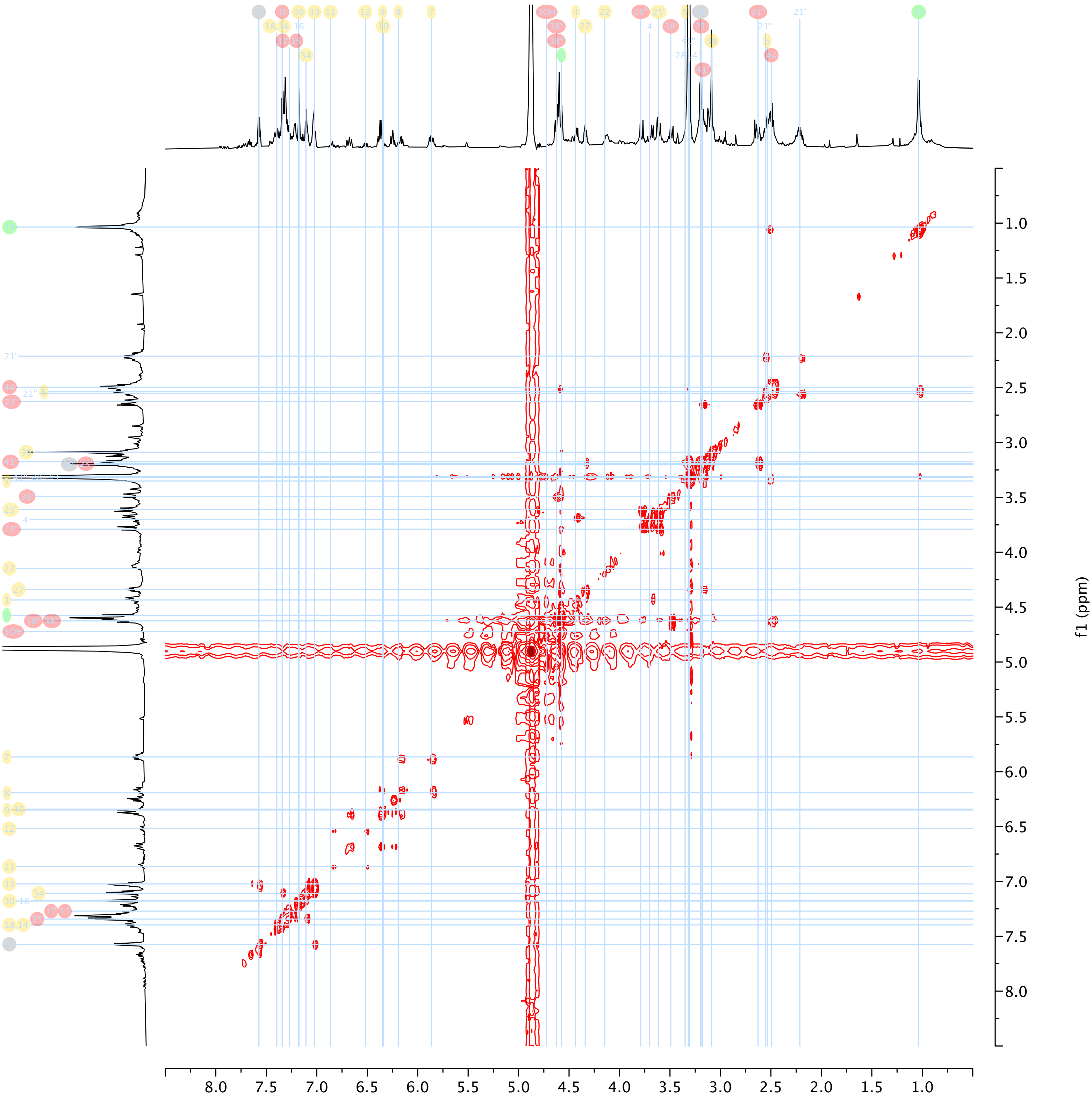

^1H-^1H-TOCSY spectrum of microsclerodermin F in CD_3OD

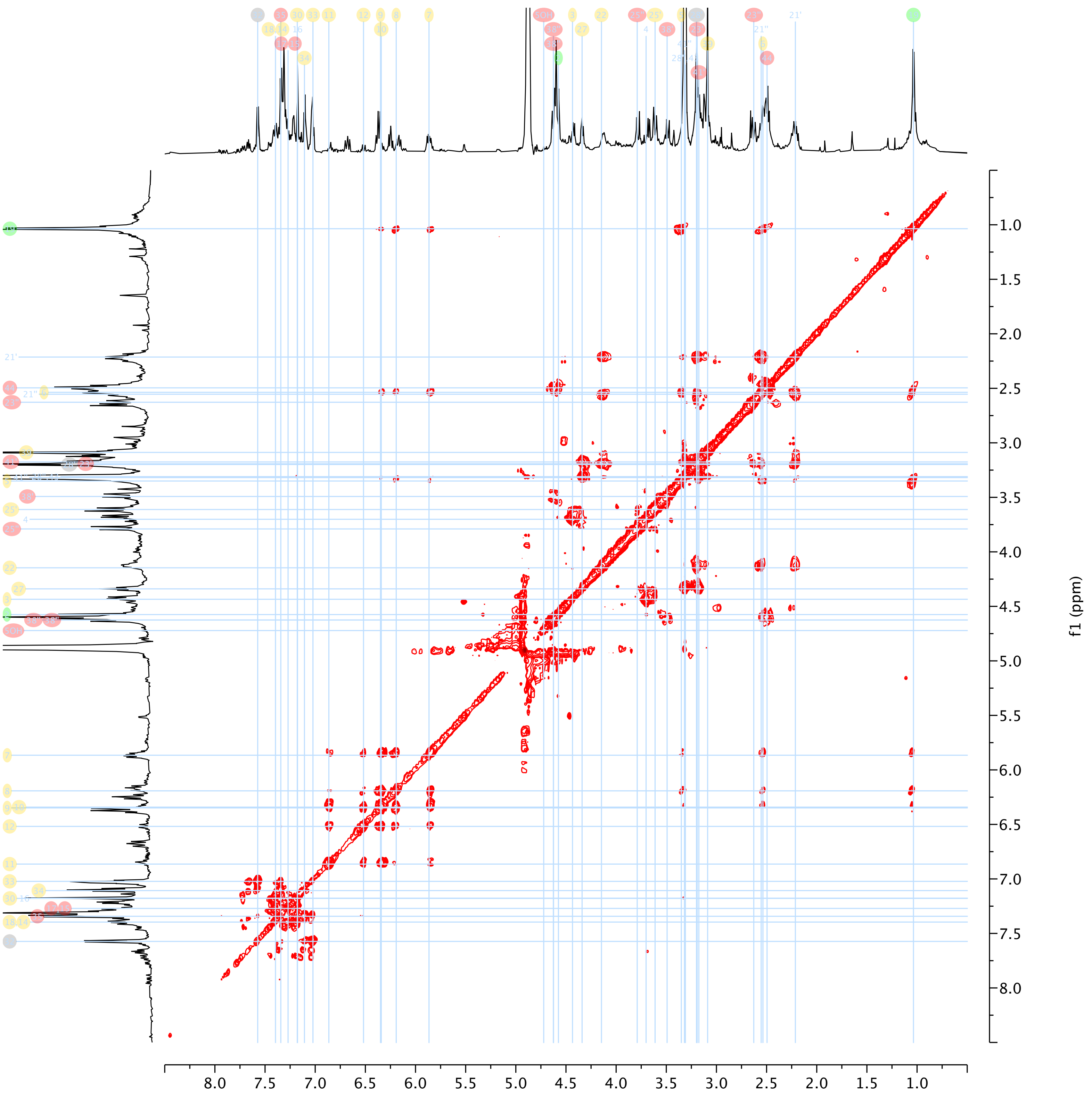

$^1H,^{13}C$-HSQC spectrum of microsclerodermin F in CD_3OD

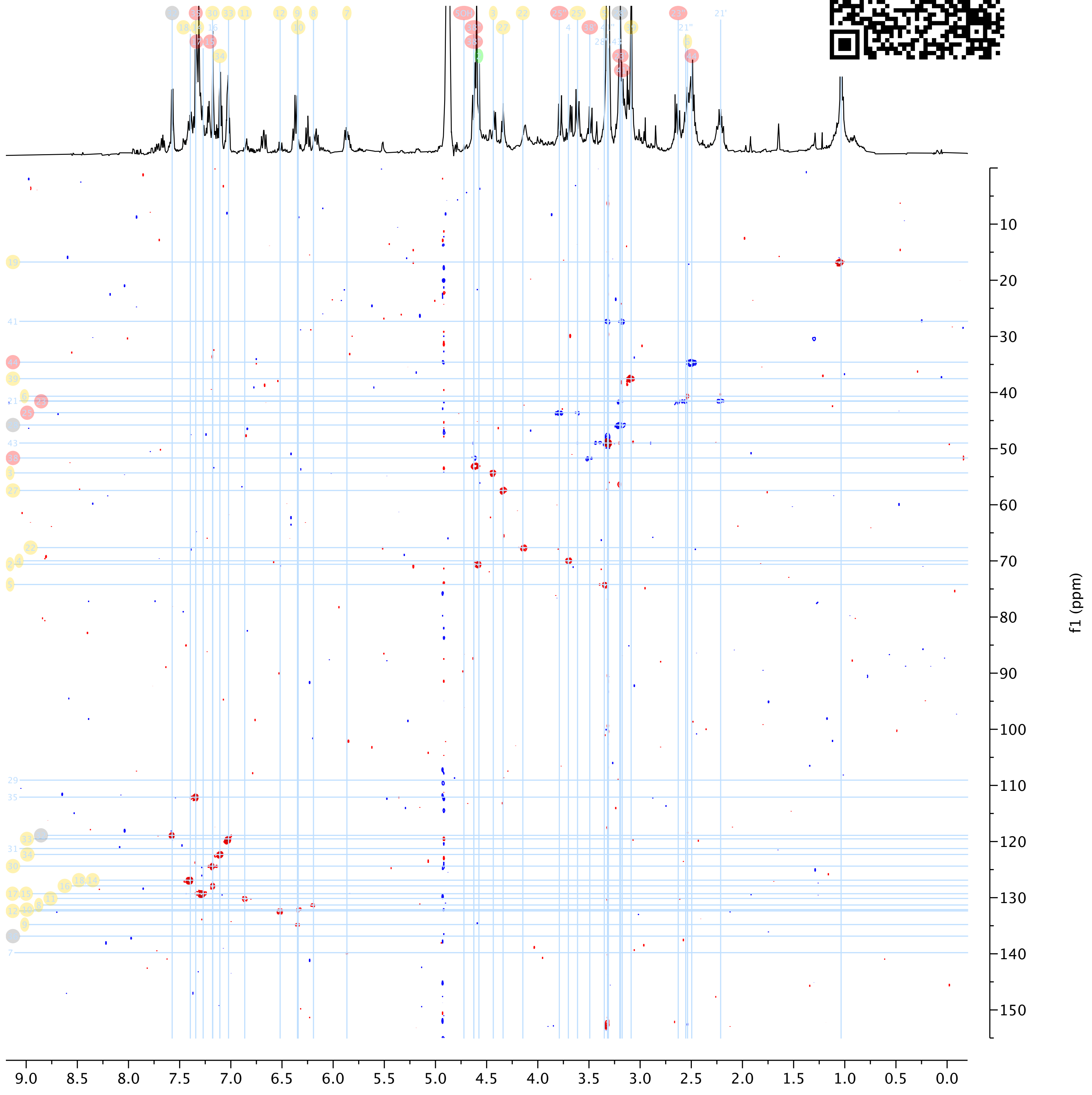

$^{1}H,^{13}C$-HSQC ASAP spectrum of microsclerodermin F in CD_3OD

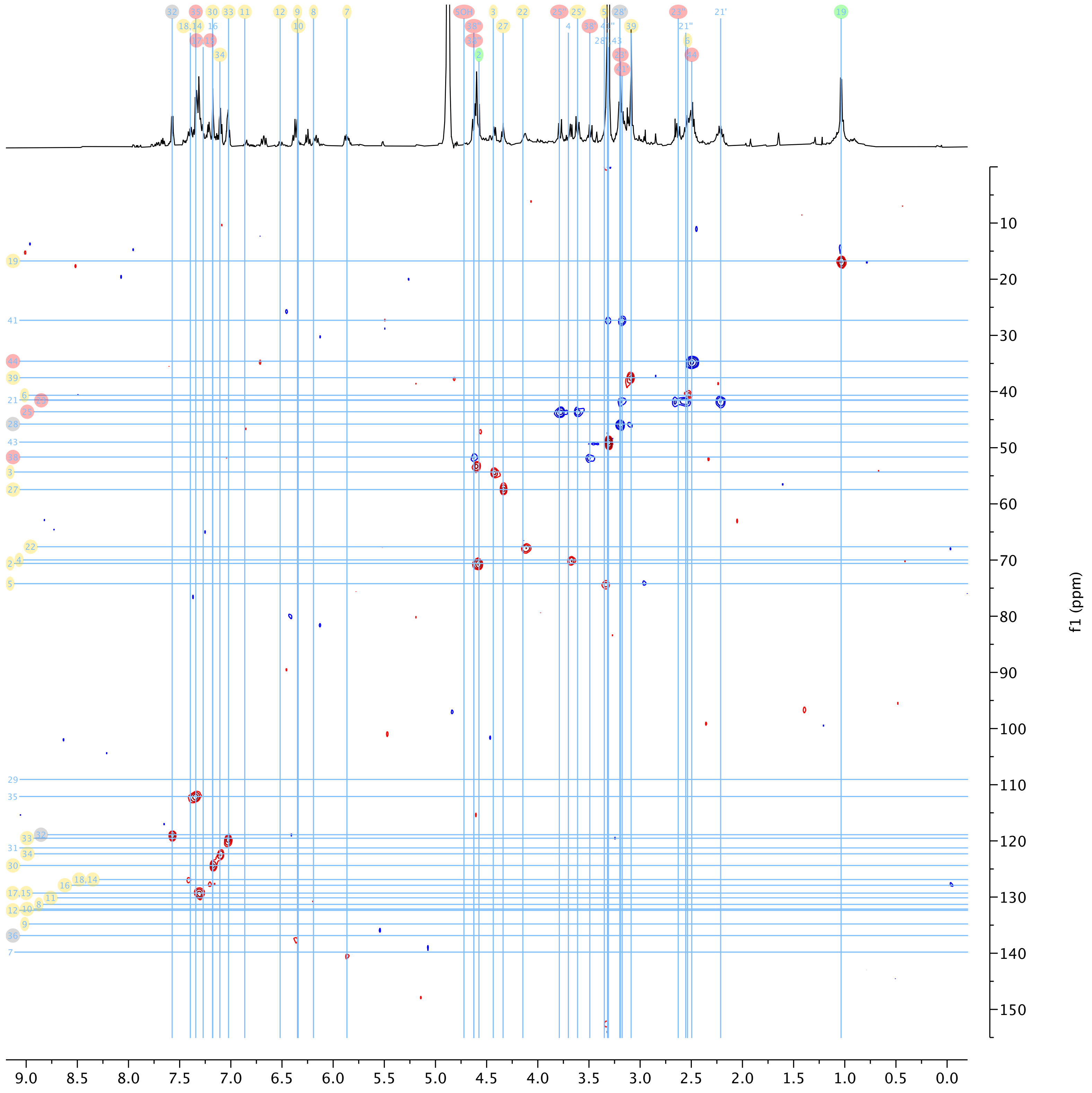

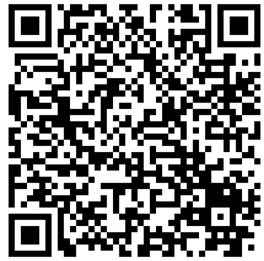

$^{1}H,^{13}C$-HMBC ASAP spectrum of microsclerodermin F in CD_3OD

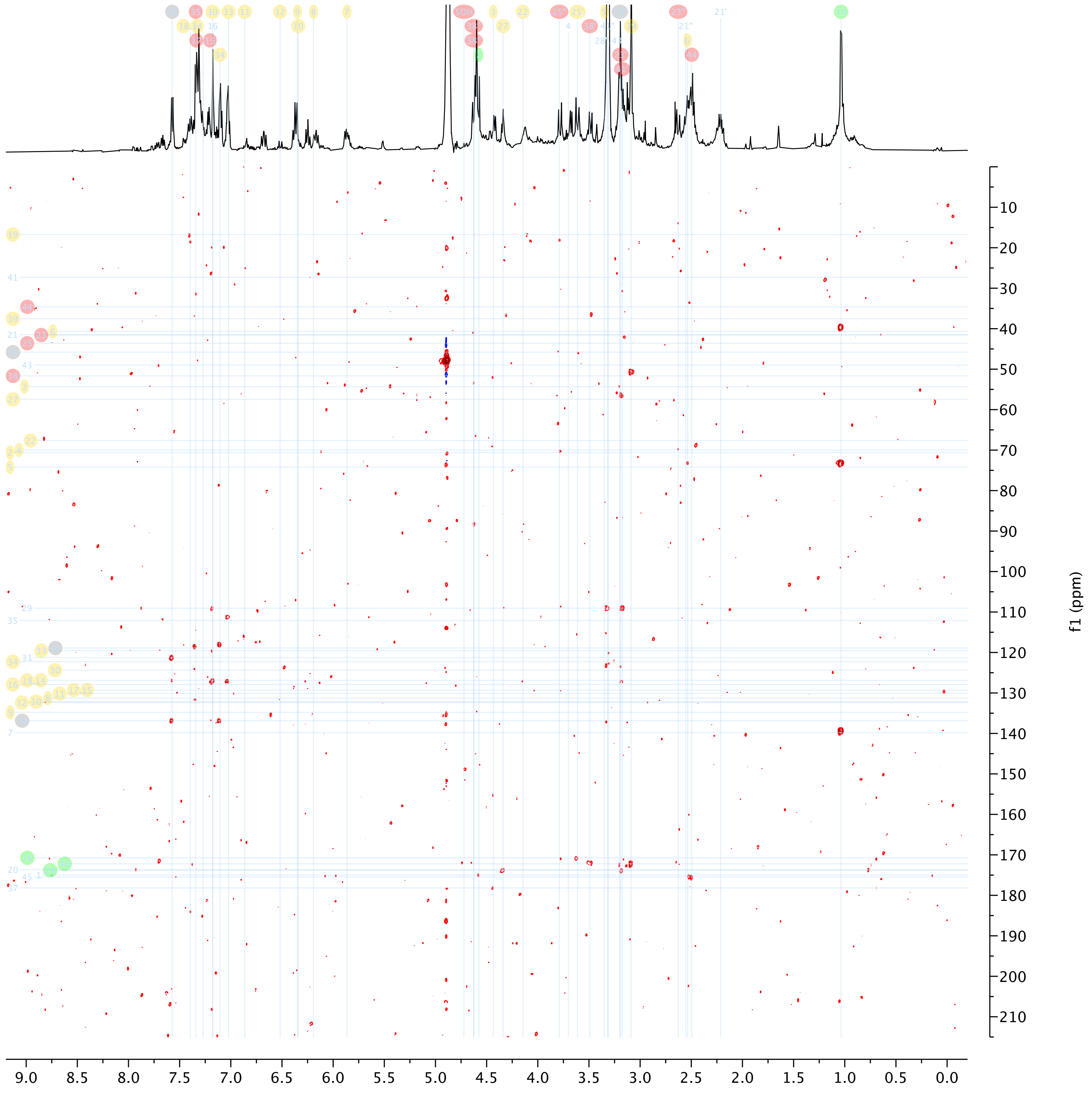

^{1}H-NMR spectrum of microsclerodermin F in DMSO-d_6

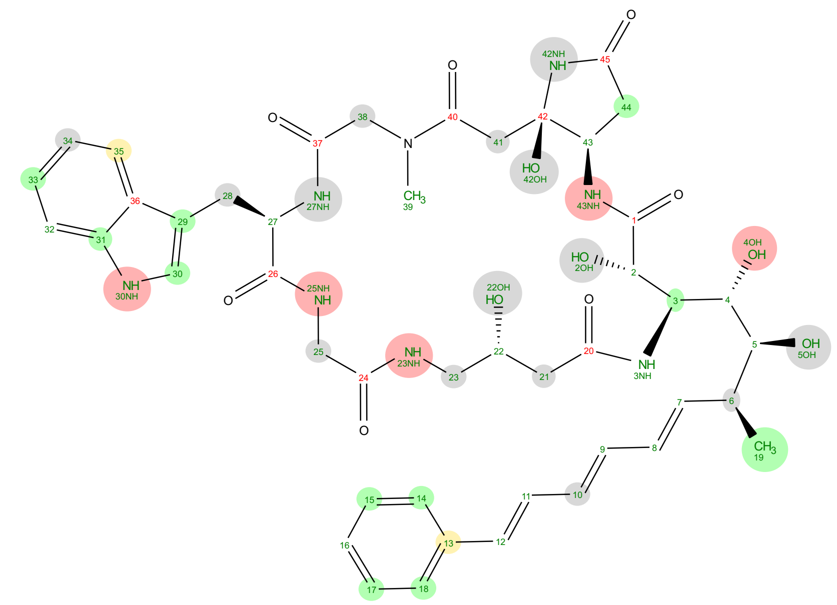

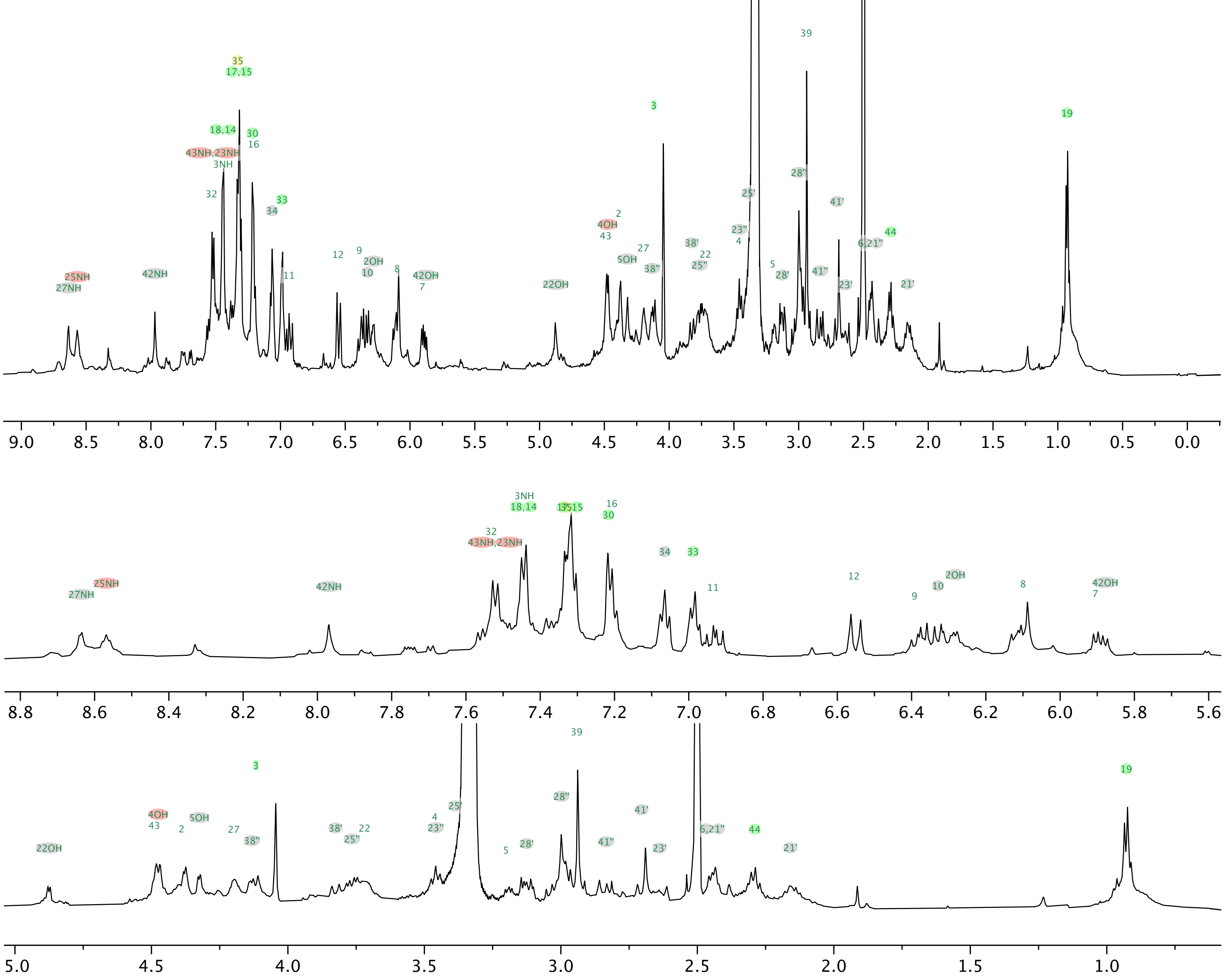

^{1}H,^{1}H-COSY spectrum of microsclerodermin F in DMSO-d_6

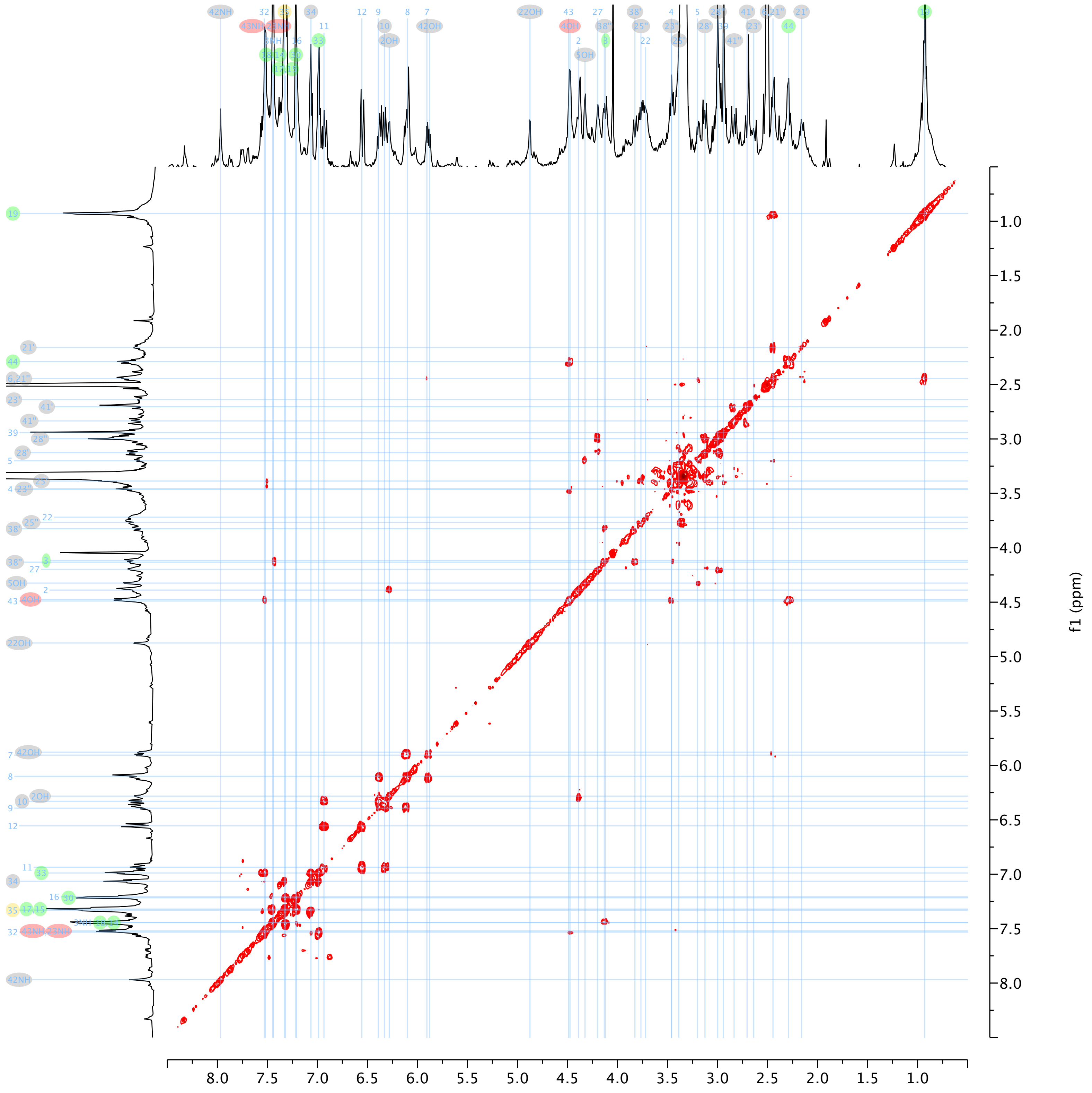

$^{1}H,^{13}C$-HSQC ASAP spectrum of microsclerodermin F in DMSO-d_6

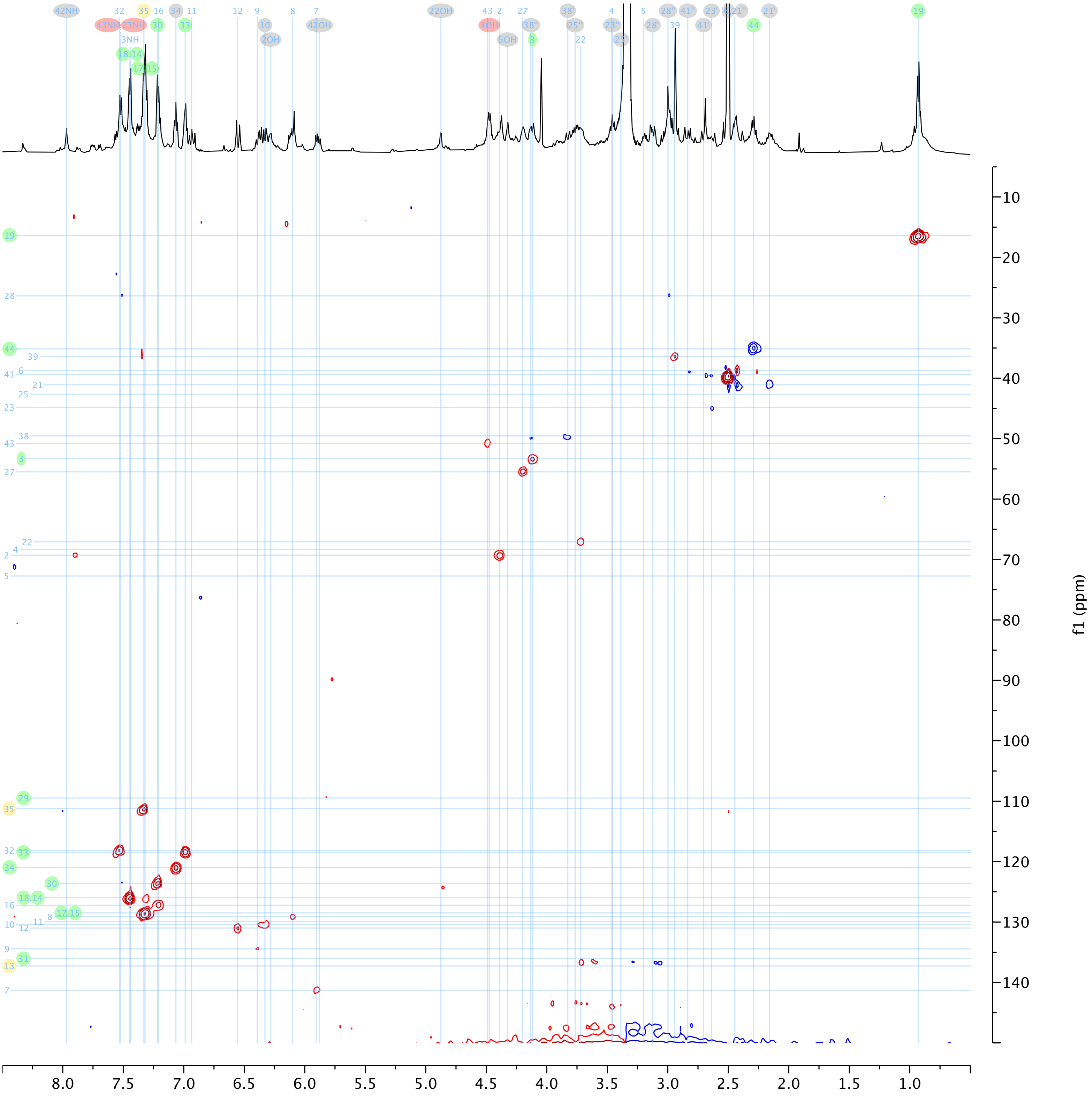

^{1}H,^{13}C-HMBC ASAP spectrum of microsclerodermin F in DMSO-d_6

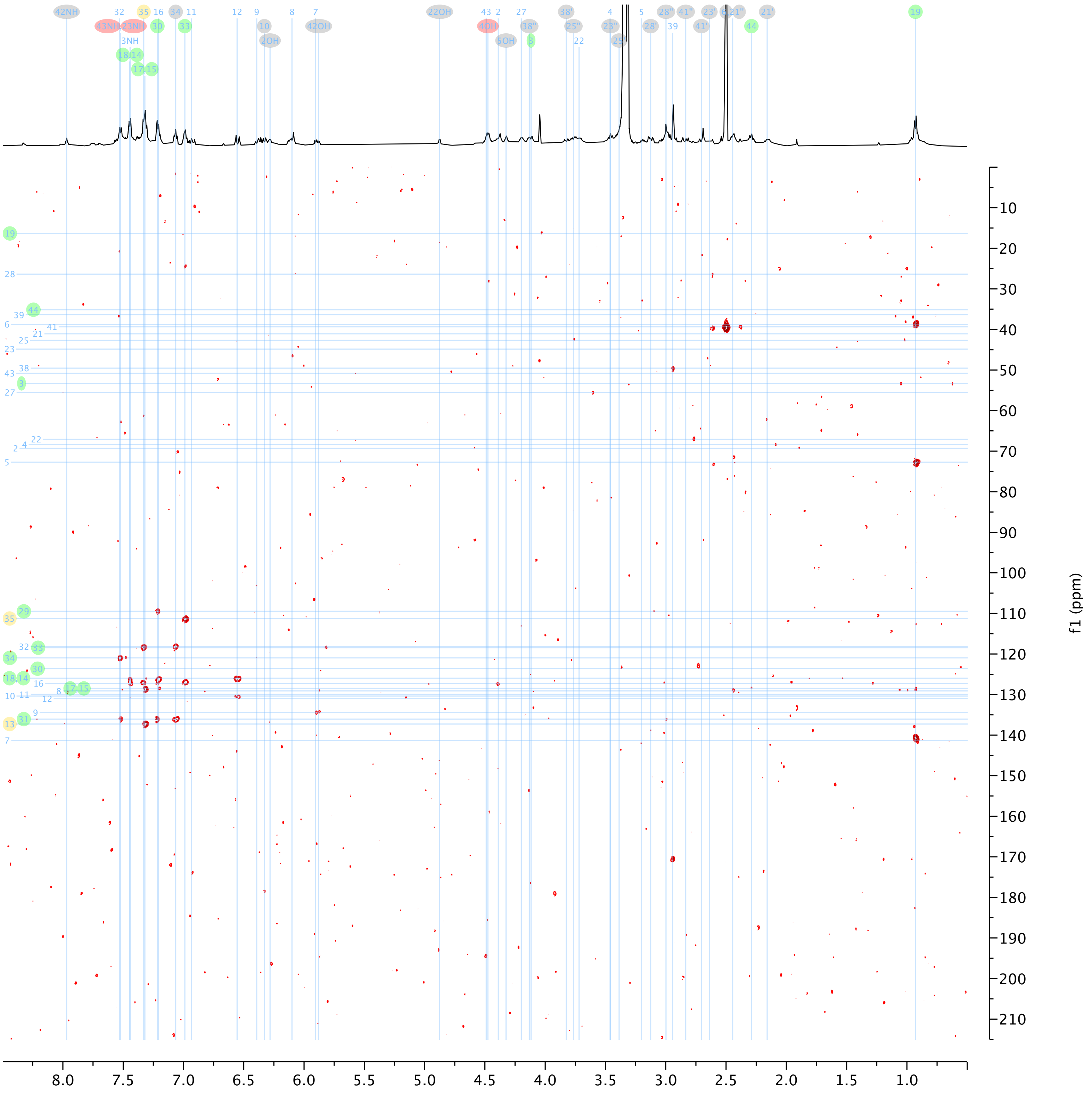

Supplement: Supplementary file 1 [file marinedrugs-23-00336-s001.zip › marinedrugs-3810785-supplementary.pdf]
